# Acid ceramidase regulates CD8+ T-cell exhaustion via type I interferon-mediated upregulation of PD-L1

**DOI:** 10.3389/fimmu.2025.1638403

**Published:** 2025-12-09

**Authors:** Zhongwen Hu, Hossam Abdelrahman, Abdelrahman Elwy, Fei Kuang, Swati Dhiman, Shafaqat Ali, Marcel Marson, Lisa Holnsteiner, Thamer A. Hamdan, Justa Friebus-Kardash, Elisa Wiebeck, Wiebke Hansen, Stefanie Scheu, Erich Gulbins, Philipp Alexander Lang, Karl Sebastian Lang, Judith Lang

**Affiliations:** 1 Institute of Immunology, University of Duisburg-Essen, Essen, Germany; 2 Institute of Medical Microbiology and Hospital Hygiene, Medical Faculty and University Hospital Düsseldorf, Heinrich Heine University Düsseldorf, Düsseldorf, Germany; 3 Department of Nephrology, University Hospital Essen, University of Duisburg-Essen, Essen, Germany; 4 Institute of Medical Microbiology, University Hospital Essen, University of Duisburg-Essen, Essen, Germany; 5 Institute of Molecular Biology, University of Duisburg-Essen, Essen, Germany; 6 Department of Surgery, University of Cincinnati, Cincinnati, OH, United States; 7 Department of Molecular Medicine II, Medical Faculty, Heinrich Heine University, Düsseldorf, Germany

**Keywords:** acid ceramidase (aCDase), ceramide, type I interferon, T cell exhaustion, programmed death ligand 1 (PD-L1), plasmacytoid dendritic cell, chronic infection, LCMV Docile

## Abstract

Besides its robust antiviral activity, type I interferon (IFN-I) also exerts immunomodulatory effects and can even drive pathology during chronic viral infections. Mechanisms that regulate IFN-I induction during virus infection, thus strongly affecting the outcome of disease, remain to be defined. Here, using the lymphocytic choriomeningitis virus (LCMV) Docile strain, we identified acid ceramidase (aCDase, *Asah1*) as a critical lipid-metabolic regulator of endosomal, nucleic acid-driven IFN-I responses and disease outcome during chronic virus infection. aCDase is highly expressed in plasmacytoid dendritic cells (pDCs) and required for robust early IFN-I production. aCDase deficiency resulted in ceramide accumulation, blunting IFN-α/β induction, impairing IFN-I-dependent upregulation of programmed death-ligand 1 (PD-L1) on antigen-presenting cells and preventing the exhaustion of virus-specific CD8^+^ T cells, leading to severe immunopathology. This pathology is abrogated by CD8^+^ T-cell depletion or by adoptive transfer of IFN-I-induced PD-L1-expressing macrophages. Conversely, limiting ceramide production in acid sphingomyelinase (Asm)-deficient mice prevented ceramide accumulation, and pDCs showed accelerated IFN-I induction. Mechanistically, ceramide abundance regulated IFN-I production by altering endosomal signaling microdomains. Collectively, our findings reveal ceramide homeostasis as a key determinant of IFN-I-driven CD8^+^ T-cell exhaustion and immunopathology during chronic viral infection and highlight aCDase as a potential therapeutic target.

## Introduction

Type I interferons (IFN-I)—primarily IFN-α and IFN-β—are the most potent innate antiviral cytokines and therefore critically dictate the course of viral infections (1, 2). IFN-I-induced effector programs target every stage of the viral life cycle, from membrane fusion to genome replication, assembly, and release. Interferon-stimulated genes (ISGs) such as Mx1/2,2′-5′-oligoadenylate synthetase (OAS), protein kinase R (PKR), and tetherin block replication, degrade viral RNA, shut down translation, or physically retain budding virions (3–6). Beyond direct antiviral activity via ISGs, IFN-I regulates innate immune functions like NK cell activation and CD8^+^ T-cell priming (7). Paradoxically, during chronic infection, this activity becomes a double-edged sword: early IFN-I limits virus spread, but sustained signaling promotes immunosuppressive mediators such as IL-10 and PD-L1, driving CD8^+^ T-cell exhaustion and preventing fatal immunopathology (8–11). How the host balances these opposing outcomes remains incompletely understood.

Plasmacytoid dendritic cells (pDCs) are the dominant source of systemic IFN-I. Their constitutively high IRF7 expression enables near-instant cytokine production upon viral sensing (12). Therefore, pDCs are a unique sentinel cell type that initiates the systemic IFN-I response after virus infection (13). pDCs sense viral nucleic acids in an endosome-rich compartment via pattern recognition receptors—including TLR3, 7, and 8 and endosomal DNA/RNA sensors—which signal through MyD88 or TRIF to activate NF-κB and IRF3/7 (14). Although endosomal receptors such as TLR3, TLR7, TLR8, and TLR9 require an acidic pH for activation (15), the cell-intrinsic factors that fine-tune pDC IFN-I output, especially in chronic infection, are still poorly defined. Identifying these regulatory nodes is essential for therapeutically shifting the balance between protection and pathology.

Ceramide, a central sphingolipid, is an emerging regulator of membrane organization and receptor signaling (16). Enrichment of ceramide in lipid rafts influences immune-receptor clustering, apoptosis, and microbial recognition (17). Perturbing ceramide homeostasis can dampen innate signaling, as shown in sphingomyelin synthase 2 (SMS2) cells, where elevated ceramide blunts TLR4 responses to LPS (18). Acid sphingomyelinase (ASM; *Smpd1*) generates ceramide by cleaving sphingomyelin, whereas acid ceramidase (aCDase; *Asah1*) hydrolyzes ceramide to sphingosine and fatty acids, thereby shaping endolysosomal lipid composition (19–22). Although pathogenic *Asah1* mutations cause Farber disease (23, 24), the impact of aCDase-controlled ceramide balance on nucleic acid-driven antiviral immunity and chronic infection remains unclear.

Here, using the chronic lymphocytic choriomeningitis virus (LCMV Docile) model, we uncover aCDase as a lipid-metabolic checkpoint that calibrates endosomal nucleic acid sensing. We show that aCDase is highly expressed in pDCs and that its absence leads to ceramide accumulation, blunted IFN-I release, impaired PD-L1 upregulation, and uncontrolled expansion of virus-specific CD8^+^ T cells, culminating in lethal immunopathology. Restoring PD-L1 on antigen-presenting cells or depleting CD8^+^ T cells rescues aCDase-deficient mice, underscoring the functional importance of this pathway. Conversely, ceramide depletion via acid sphingomyelinase (Smpd1) deficiency amplifies IFN-I and inflammatory cytokines. Our findings identify ceramide balance, maintained by aCDase, as a key determinant of the IFN-I–PD-L1 axis and immune homeostasis during persistent viral infection.

## Results

### aCDase in pDCs is required for an early systemic IFN-I burst during LCMV infection

**Figure 1:**
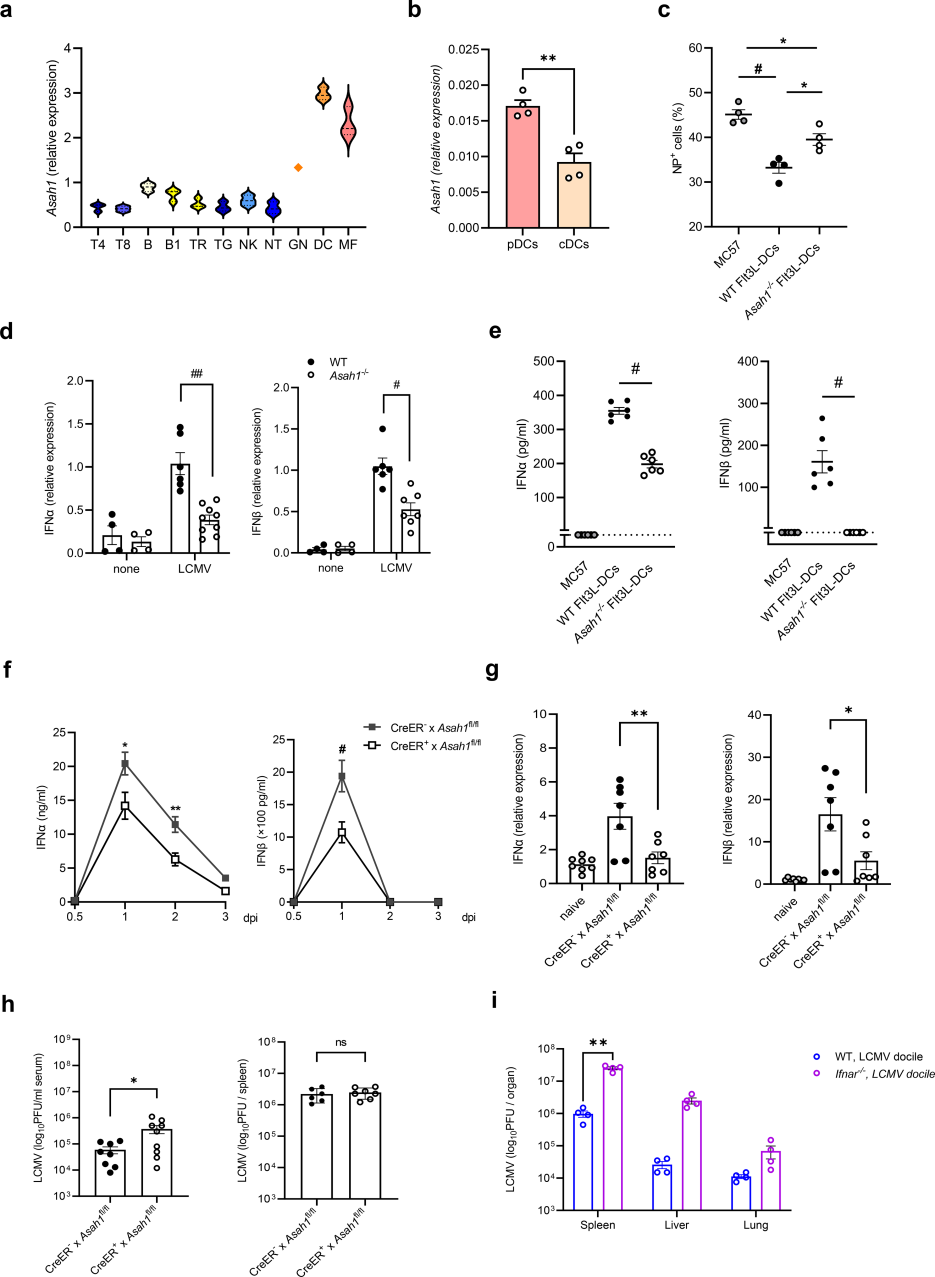
aCDase in pDCs is required for efficient IFN-I expression after LCMV Docile infection. **(a)** Relative expression of *Asah1* gene in mouse cells using raw microarray data from GSE75202. The list of all Gene Expression Omnibus accession numbers and corresponding cell populations and series is available in **Supplementary Table S1** in the Supplementary Material. **(b)** Expression of *Asah1* in pDCs and cDCs determined by quantitative real-time polymerase chain reaction (qRT-PCR; *n* = 4; unpaired Student’s *t*-test). **(c)** Infected cells as determined by LCMV nucleoprotein^+^ (NP^+^) cells by fluorescence-activated cell sorting (FACS) analyses of LCMV Docile-infected [multiplicity of infection (MOI) 1] MC57 fibroblasts without coculture (MC57) or cocultured with WT Flt3L-DCs (WT DCs) and *Asah1*^−/−^ Flt3L-DCs (*Asah1*^−/−^ DCs) for 48 h [*n* = 4; one-way ANOVA (Tukey’s multiple comparison)]. **(d)** Relative IFN-α and IFN-β expression determined by qRT-PCR 24 h post-infection (h.p.i.) of Flt3L-DCs from WT and *Asah1*^−/−^ mice infected with LCMV Docile at MOI 1 [*n* = 4–9; two-way ANOVA (Sidak’s multiple comparison)]. **(e)** MC57 fibroblasts infected with LCMV Docile at MOI 1 MC57 or co-cultured with WT Flt3L-DCs (WT DCs) and *Asah1*^−/−^ Flt3L-DCs (*Asah1*^−/−^ DCs) for 48 h IFN-α and IFN-β in supernatant were detected by enzyme-linked immunosorbent assay [ELISA; *n* = 6; one-way ANOVA (Tukey’s multiple comparison)]. **(f, g)** Serum IFN-α/β concentrations measured by ELISA at the indicated timepoints **(g)** of spleens determined by qRT-PCR 3 d.p.i. of tamoxifen-induced CreER^+^ × *Asah1*^fl/fl^ and CreER^−^ × *Asah1*^fl/fl^ mice infected with 2 × 10^6^ PFU LCMV Docile [**(f)***n* = 5–9; two-way ANOVA [Sidak’s multiple comparison]; **(g)***n* = 7–8; one-way ANOVA (Tukey’s multiple comparison)]. **(h)** LCMV plaque assay of serum and spleen at 3 d.p.i. from tamoxifen-induced CreER^+^ × *Asah1*^fl/fl^ and CreER^−^ × *Asah1*^fl/fl^ infected with 2 × 10^6^ PFU LCMV Docile (*n* = 8–9; unpaired Student’s *t*-test). **(i)** LCMV plaque assay of the spleen, liver, and lung at 3 d.p.i. from wild-type (WT) and *Ifnar*^−/−^ mice infected with 2 × 10^6^ PFU LCMV Docile (*n* = 4; two-way ANOVA [Sidak’s multiple comparison]). In **(b–e)**, three independent experiments; in **(f–i)**, two to three independent experiments. All data are shown as mean ± SEM. *p ≤ 0.05, **p ≤ 0.01, #*p* ≤ 0.001, and ##*p* ≤ 0.0001. ns, not significant.

We previously demonstrated that sphingosine produced by aCDase in intralumenal vesicles (ILVs) of multivesicular bodies (MVBs) is essential for the control of herpes simplex virus type 1 (HSV-1) in macrophages (25). In order to investigate the potential function of aCDase in other immune cells, we analyzed the relative expression of *Asah1* in 11 different immune cell populations from a previously published dataset on the Gene Expression Omnibus (GEO) database. We found that *Asah1* has the highest expression in dendritic cells (DC; splenic CD11c^+^MHCII^+^ Fms-related tyrosine kinase 3 ligand [Flt3L]^+^) (**Figure 1a**, **Supplementary Table 1**). Next, we cultured DCs using Flt3 ligand and sorted conventional DCs (cDCs) and plasmacytoid dendritic cells (pDCs; **Supplementary Figure 1**). The expression of *Asah1* was analyzed by qRT-PCR and revealed higher expression in pDCs than in cDCs (**Figure 1b**).

pDCs are key players in the early type I IFN (IFN-I; IFN-α/β) production in response to several viral infections (26). We therefore hypothesized that *Asah1* may play a role in type I IFN regulation. As pDCs were reported to be rather non-permissive to viral infections, but reactive to infected cells upon physical contact (27), we cocultured LCMV Docile-infected MC57 cells with Flt3L-DCs. While coculture with WT Flt3L-DCs yielded fewer infected cells compared to MC57 culture alone, coculture with *Asah1*^−/−^ Flt3L-DCs could not reduce viral loads to the same extent (**Figure 1c**). Interestingly, the expression of IFN-α and IFN-β in Flt3L-DCs was increased after LCMV Docile infection, but significantly higher in WT rather than *Asah1*^−/−^ Flt3L-DCs (**Figure 1d**). As differences upon direct infection of non-permissive pDCs were rather small, we again performed cocultures with MC57 cells. The IFN-α/β production in response to LCMV Docile infection of MC57 was below the detection limit, WT Flt3L-DCs produced high levels of IFN-α/β, while in *Asah1*^−/−^ Flt3L-DCs, IFN-α/β levels were significantly lower (**Figure 1e**). This tremendous effect was apparent in the coculture setting rather than in a direct *in-vitro* infection, indicating that intercellular sensing triggered by infected MC57 cells—rather than cell-intrinsic replication—might be the dominant stimulus in this process.

To assess the *in-vivo* relevance of aCDase, we infected tamoxifen-treated inducible aCDase-deficient mice using the common ER Cre model (CreER × *Asah1*^fl/fl^) with LCMV Docile intravenously (i.v.), and IFN-α/β expression in serum was detected by enzyme-linked immunosorbent assay (ELISA). Compared to CreER^−^ × *Asah1*^fl/fl^, IFN-α levels in serum of CreER^+^ × *Asah1*^fl/fl^ mice were significantly lower at 1 and 2 days post-infection (d.p.i.; **Figure 1f**). Likewise, the amount of IFN-β in serum peaked at 1 d.p.i. and was significantly lower in CreER^+^ × *Asah1*^fl/fl^ than in CreER^−^ × *Asah1*^fl/fl^ mice (**Figure 1f**). At 3 d.p.i., we detected the mRNA levels of IFN-α and IFN-β in the spleens by quantitative real-time polymerase chain reaction (qRT-PCR), which were significantly lower in CreER^+^ × *Asah1*^fl/fl^ mice (**Figure 1g**). Therefore, we wondered whether the lower IFN response in CreER^+^ × *Asah1*^fl/fl^ mice would contribute to early replication of the chronic virus. We detected the viral loads in the blood and spleen of CreER^−^ × *Asah1*^fl/fl^ and CreER^+^ × *Asah1*^fl/fl^ mice 3 d.p.i. using the plaque assay (**Figure 1h**). While a slight difference between CreER^−^ × *Asah1*^fl/fl^ and CreER^+^ × *Asah1*^fl/fl^ mice was detected in the serum, there was no difference in tissues like the spleen (**Figure 1h**), and we found that the virus titer was significantly increased in the splenic tissue of IFNAR^−/−^ mice lacking type I interferon receptor function (**Figure 1i**).

In conclusion, aCDase is highly expressed in pDCs, and its deficiency correlates with the reduced production of IFN-α and IFN-β, whereas early viral replication is unaffected *in vivo*. As endosomal nucleic acid sensors are potent triggers of IFN-I production, we hypothesized that the ceramide accumulation seen in *Asah1^−^/^−^* pDCs might impede these signaling pathways and account for the diminished interferon response.

### Ceramide abundance inversely regulates endosomal nucleic acid-driven IFN-I

**Figure 2:**
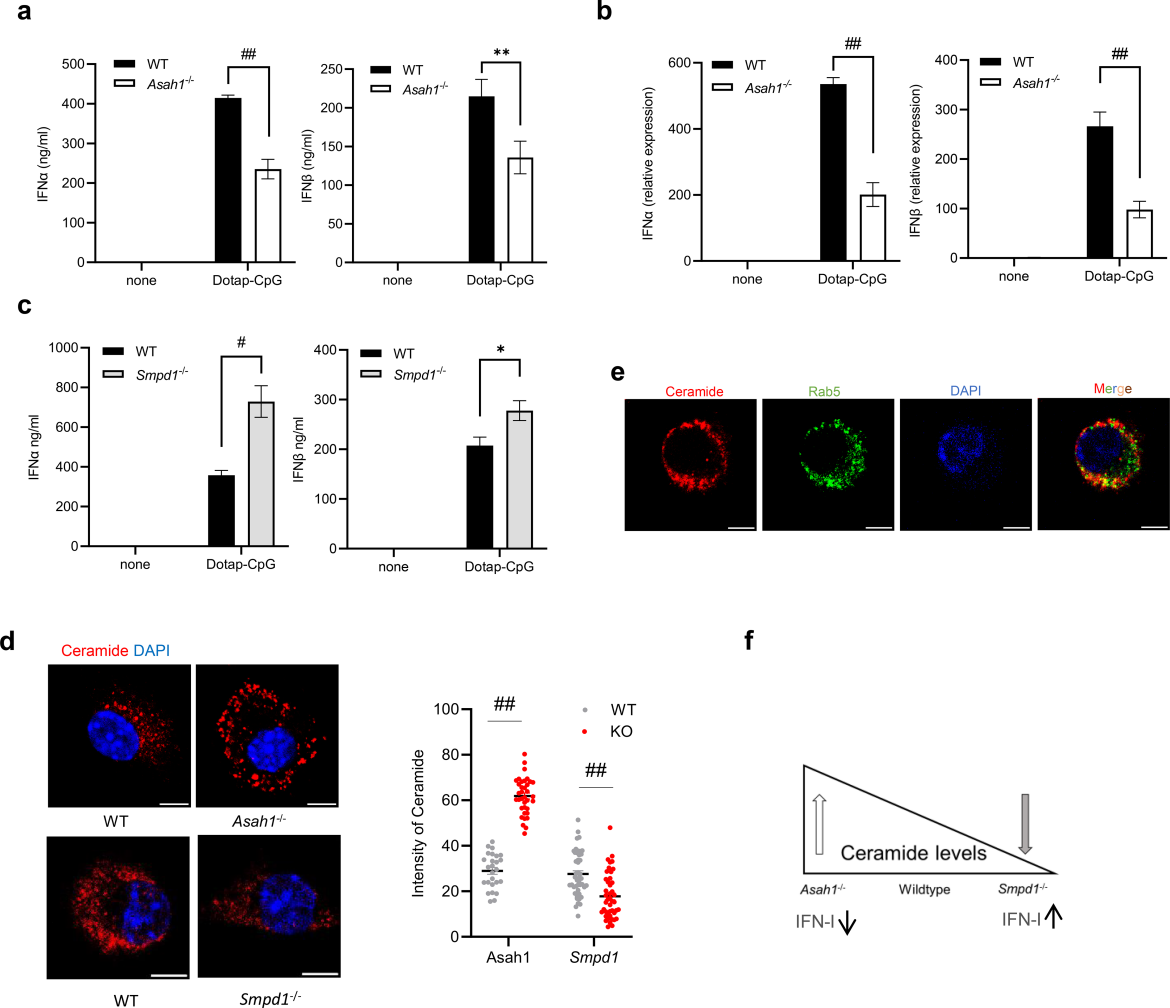
Ceramide balance bidirectionally tunes endosomal nucleic acid-driven type I IFN. **(a, b)** Enzyme-linked immunosorbent assay [ELISA, **(a)** 16 h] or relative IFN-α/β expression by quantitative RT-PCR [qRT-PCR, **(b)** 24 h] in cells from wild-type (WT) or *Asah1*^^-^/^-^^ Flt3L-DC cultures stimulated with PBS or Dotap-CpG [*n* = 4–8; two-way ANOVA (Sidak’s multiple comparison)]. **(c)** ELISA for IFN-α and IFN-β at 16 h of supernatants from WT or *Smpd1^−/−^* Flt3L-DCs stimulated with Dotap-CpG (*n* = 12–14; unpaired Student’s *t*-test). **(d)** Confocal image (left) and analysis of intensity by ImageJ (right) of WT or *Asah1*^−/−^ and Smpd1^-^/^-^ Flt3L-DCs fixed and stained with anti-ceramide antibody. Scale bar: 5 µm. Ceramide staining intensity quantified using ImageJ software from approximately 10 cells per image; three images from each experiment were quantified [*n* = 26–36; two-way ANOVA (Sidak’s multiple comparison)]. **(e)** Dotap-CpG stimulated WT Flt3L-DCs fixed after 3 h and stained for ceramide, Rab5, and DAPI. Scale bar: 5 µm. **(f)** Diagram illustrating ceramide levels in *Asah1*^−/−^, WT, and *Smpd1^−/−^* pDCs. All data are shown as mean ± SEM. *p ≤ 0.05, **p ≤ 0.01, #*p* ≤ 0.001, and ##*p* ≤ 0.0001.

Numerous studies have demonstrated that stimulation of Flt3L-derived dendritic cells (Flt3L-DCs) with DOTAP-CpG complexes activates endosomal nucleic acid sensors, leading to robust induction of type I interferon (IFN-I; IFN-α/β) (28). To determine whether aCDase regulates this IFN-I response, we cultured WT and *Asah1*^−/−^ Flt3L-DCs and stimulated them with DOTAP-CpG. Indeed, *Asah1*^−/−^ DCs produced significantly lower amounts of IFN-α and IFN-β protein and reduced IFN-α or IFN-β transcripts compared to WT DCs (**Figures 2a, b**), confirming a critical role of aCDase in regulating IFN-I production. Conversely, DCs deficient for acid sphingomyelinase (Asm; *Smpd1^-^/^-^*), which normally catalyzes the hydrolysis of sphingomyelin to ceramide, thereby increasing the cellular ceramide content (29), produced significantly more IFN-α/β protein upon DOTAP-CpG stimulation (**Figure 2c**), suggesting a negative regulatory role for ceramide.

To investigate whether ceramide abundance correlates with this altered IFN-I response, we next quantified basal ceramide levels using confocal microscopy. Baseline ceramide fluorescence intensity was significantly elevated in *Asah1*^−/−^ DCs and notably lower in *Smpd1^−/−^* DCs compared to WT (**Figure 2d**), establishing an inverse correlation between cellular ceramide levels and the magnitude of IFN-I response.

Confocal imaging of WT Flt3L-DCs at 3 h post-CpG stimulation revealed strong colocalization of ceramide with Rab5-positive endosomes (**Figure 2e**), consistent with the known role of ceramide in regulating signaling within these endosomal compartments. In contrast, ceramide distribution was aberrantly clustered at the cell periphery in *Asah1*^−/−^ DCs, as reflected quantitatively (**Figure 2d**).

We then extended our analysis to other endosomal nucleic acid agonists. Stimulation of BMDCs with Resiquimod (R848, TLR7/8 ligand) or poly(I:C) (TLR3 ligand) revealed a similar inverse pattern of cytokine secretion: TNF-α and IL-6 production was significantly reduced in *Asah1*^−/−^ cells yet significantly increased in *Smpd1^−/−^* cells compared to WT (**Supplementary Figure S2b**). Additionally, normal uptake and trafficking of nucleic acid ligands into endosomes was confirmed by colocalization experiments using fluorescently labeled CpG-FITC and endosome-labeled dextran (**Supplementary Figure S2c**).

Western blot analysis further demonstrated that phosphorylation of IRF5 and IRF7, central mediators downstream of endosomal nucleic acid sensing, was markedly delayed and reduced in *Asah1*^−/−^ DCs compared to WT cells (**Supplementary Figure S2d**). Finally, production of IL-10, IL-1β, and IL-12p70 cytokines remained unchanged irrespective of *Asah1* or Smpd1 deficiency, highlighting specificity in ceramide-regulated signaling pathways (**Supplementary Figure S2e**).

Taken together, our data demonstrate that ceramide functions as a crucial regulator of IFN-I and related pro-inflammatory cytokines produced by endosomal nucleic acid sensors. Elevated ceramide levels (as in *Asah1^−/−^* DCs) profoundly diminish IFN-I and pro-inflammatory cytokine responses, whereas reduced ceramide (as in *Smpd1^−/−^* DCs) significantly enhances these responses. A schematic representation summarizing the inverse relationship between ceramide abundance and IFN-I production is provided in **Figure 2f**.

### aCDase orchestrates PD-L1 expression through IFN-I upregulation

**Figure 3:**
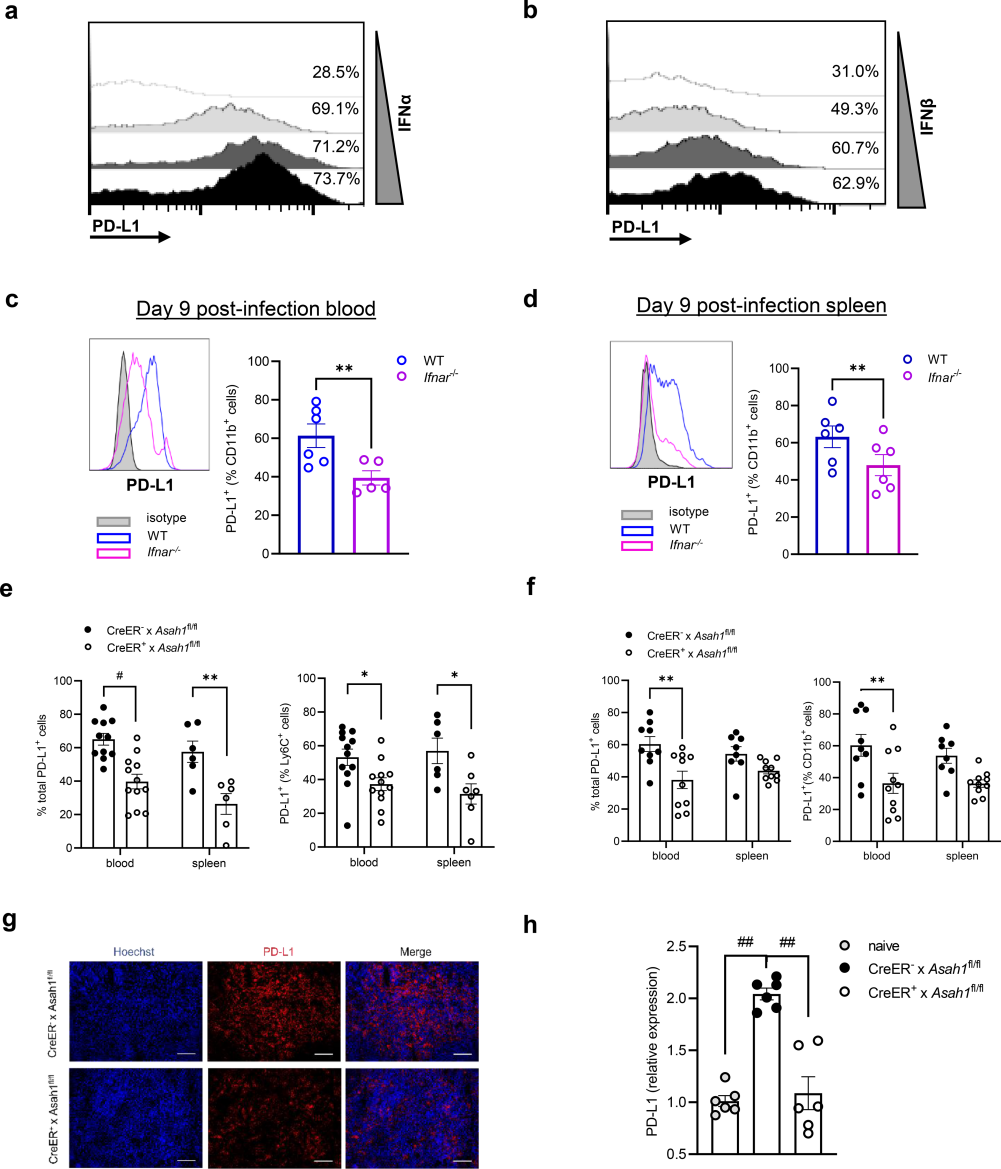
aCDase orchestrates PD-L1 expression through IFN-I upregulation. **(a, b)** M-CSF-induced bone marrow-derived macrophages (BMDMs) from C57BL/6 wild-type (WT) mice were treated by recombinant mouse IFN-α **(a)** and IFN-β **(b)** protein with 0, 100, 500, and 1,000 U/mL for 24 h Representative histogram from flow cytometry of PD-L1 (CD274^+^) surface expression. **(c, d)** PD-L1 expression (left) and mean fluorescence intensity (right) of blood **(c)** and spleen **(d)** of 2 × 10^6^ PFU LCMV Docile-infected WT and *Ifnar^−^*^/−^ mice 9 days post-infection (d.p.i.; *n* = 5–6; paired Student’s *t*-test). **(e, f)** Percentage of PD-L1-positive cells in lymphocytes and Ly6C^+^ cells on day 3 **(e)** and percentage of PD-L1-positive cells in lymphocytes and CD11b^+^ cells on day 8 **(f)** from the blood (left) and spleen (right) of tamoxifen-induced CreER^+^ × *Asah1*^fl/fl^ and CreER^−^ × *Asah1*^fl/fl^ mice infected with 2 × 10^6^ PFU LCMV Docile strain and sacrificed on 3 d.p.i. **(e)**; [*n* = 6–12; two-way ANOVA (Sidak’s multiple comparison)] and 8 d.p.i. **(f)**; [*n* = 8–10; two-way ANOVA (Sidak’s multiple comparison)], respectively. **(g, h)** Histology of spleens **(g)** and relative expression in quantitative real-time polymerase chain reaction (qRT-PCR; **h**) from tamoxifen-induced CreER^+^ × *Asah1*^fl/fl^ and CreER^−^ × *Asah1*^fl/fl^ mice infected with 2 × 10^6^ PFU LCMV Docile strain and sacrificed on 3 d.p.i. [*n* = 6; one-way ANOVA (Tukey’s multiple comparison)]. Normalized to naive C57BL/6 mice. Scale bar: 100 µm. All data are shown as mean ± SEM. *p ≤ 0.05, **p ≤ 0.01, #*p* ≤ 0.001, and ##*p* ≤ 0.0001. In **(a–d)** and **(g, h)**, three independent experiments. In **(e, f)**, four independent experiments.

Programmed death-ligand 1 (PD-L1) suppresses excessive T-cell activity during chronic viral infections (30). Because IFN-I is a well-established inducer of PD-L1 (31), we first confirmed dose responsiveness *in vitro*. Treating bone marrow-derived macrophages (BMDMs) and dendritic cells (BMDCs and Flt3L-DCs) with increasing concentrations of IFN-α or IFN-β for 24 h caused a robust, concentration-dependent rise in PD-L1 surface expression (**Figures 3a, b**, **Supplementary Figures 3a, b**).

To verify this relationship *in vivo*, we infected WT and *Ifnar^−^/^−^* mice with LCMV Docile. At 9 d.p.i., *Ifnar^−^/^−^* mice displayed markedly lower PD-L1 expression on CD11b^+^ myeloid cells in both blood and spleen compared with WT controls (**Figures 3c, d**), confirming the dominant role of IFN-I in PD-L1 regulation during infection.

We next asked whether impaired IFN-I production in aCDase-deficient mice limits PD-L1 induction. Tamoxifen-treated CreER^+^ × *Asah1*^fl/fl^ and CreER^−^ × *Asah1*^fl/fl^ littermates were infected with LCMV Docile and analyzed 3 d.p.i. PD-L1 expression on lymphocytes and Ly6C^+^ early macrophages was significantly reduced in CreER^+^ × *Asah1*^fl/fl^ mice relative to CreER^−^ × *Asah1*^fl/fl^ controls (**Figure 3e**). This deficit persisted: at 8 d.p.i., PD-L1 remained lower on lymphocytes and CD11b^+^ late macrophages in CreER^+^ mice (**Figure 3f**). Immunohistochemistry corroborated diminished splenic PD-L1 protein at 8 d.p.i. (**Figure 3g**), and qRT-PCR confirmed reduced Pdl1 transcripts in CreER^+^ spleens at 3 d.p.i. (**Figure 3h**).

To further assess responder-cell heterogeneity, we stimulated BMDMs and BMDCs derived from tamoxifen-treated CreER^+^ × *Asah1*^fl/fl^ and CreER^−^ × *Asah1*^fl/fl^ littermates with increasing concentrations of IFN-β (0–1,000 U/mL). Both cell types displayed a clear dose-dependent PD-L1 upregulation (**Supplementary Figure 3c**). However, CreER^+^ × *Asah1*^fl/fl^ BMDCs reached significantly lower maximal PD-L1 levels compared to control (CreER^−^ × *Asah1*^fl/fl^) BMDCs, while CreER^+^ × *Asah1*^fl/fl^ BMDMs showed slightly increased PD-L1 induction. These findings not only confirm cell-type-specific IFN-I responsiveness but also suggest an intrinsic role for altered ceramide distribution in modulating PD-L1 surface expression independently of IFN-I.

Collectively, these findings establish that aCDase is essential for IFN-I-driven PD-L1 expression on antigen-presenting cells, linking ceramide-controlled IFN-I production to checkpoint–ligand induction during chronic viral infection.

### Reduced expression of PD-L1 in aCDase-deficient mice leads to CD8^+^ T-cell hyperactivation

**Figure 4:**
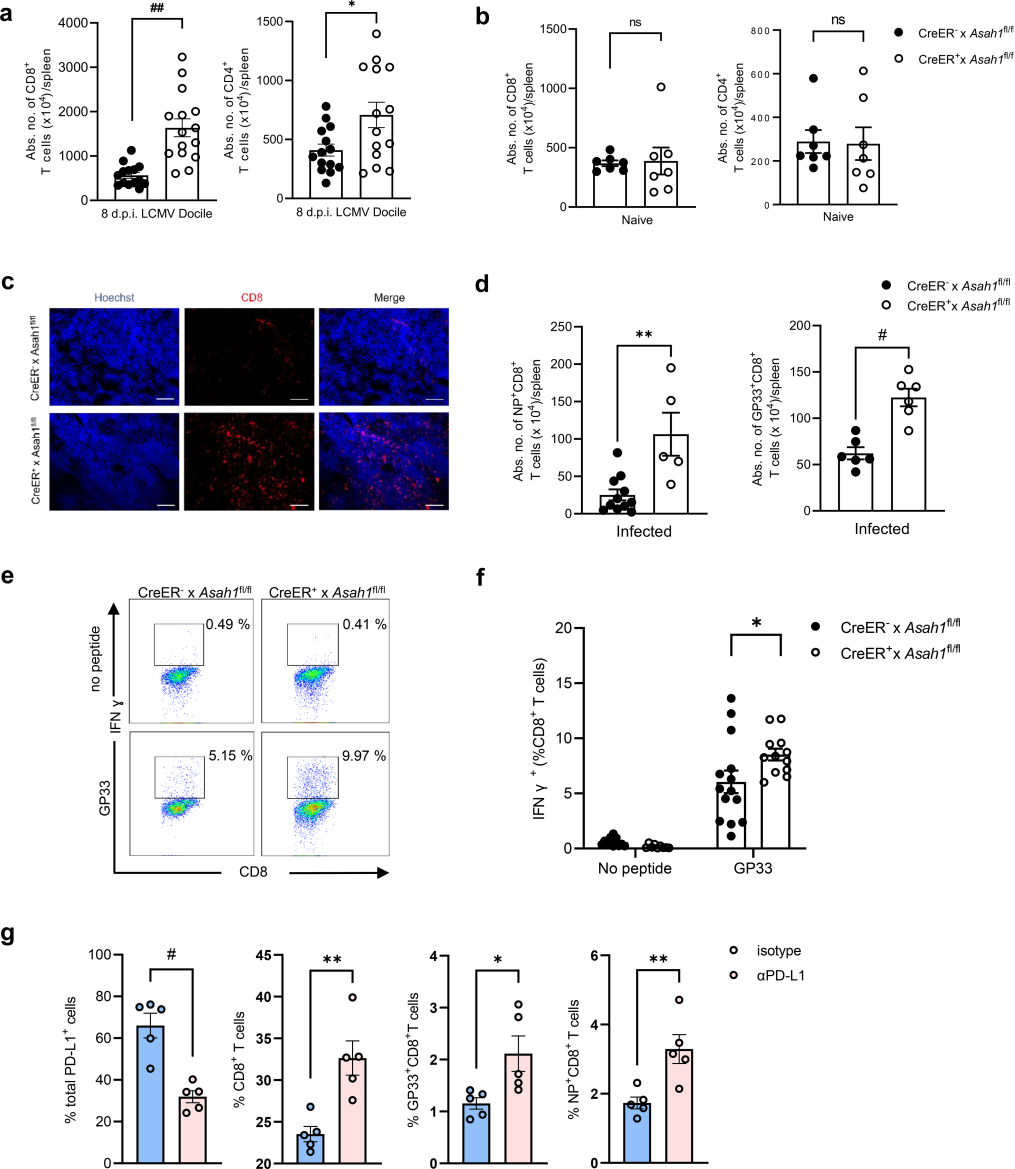
Reduced expression of PD-L1 in aCDase-deficient mice leads to CD8^+^ T-cell hyperactivation. **(a–c)** Flow cytometry analysis of CD8^+^ and CD4^+^ T cells in spleens quantified by counting beads of tamoxifen-induced CreER^+^ × *Asah1*^fl/fl^ and CreER^−^ × *Asah1*^fl/fl^ mice prior to infection **(b)**; *n* = 7; unpaired Student’s *t*-test) or 8 days post-infection (d.p.i.; A; *n* = 14–15; unpaired Student’s *t*-test) with 2 × 10^4^ PFU LCMV Docile and histology of the spleens thereof **(c)**. Scale bar: 100 µm. **(d)** LCMV-specific splenic NP^+^ (left) or GP33^+^ (right) CD8^+^ T of tamoxifen-induced CreER^+^ × *Asah1*^fl/fl^ and CreER^−^ × *Asah1*^fl/fl^ mice 8 d.p.i. with 2 × 10^4^ PFU LCMV Docile (*n* = 5–11; unpaired Student’s *t*-test). **(e, f)** Representative dot plots **(e)** and frequency **(F)** of IFN-γ^+^CD8^+^ T cells restimulated with GP33 peptide or unstimulated of tamoxifen-induced CreER^+^ × *Asah1*^fl/fl^ and CreER^−^ × *Asah1*^fl/fl^ mice 8 d.p.i. with 2 × 10^4^ PFU LCMV Docile (*n* = 9–14; two-way ANOVA [Sidak’s multiple comparison]). **(g)** Percentage of PD-L1-positive cells in lymphocytes and percentage of CD8^+^ T cells, GP33^+^CD8^+^ T cells, and NP^+^CD8^+^ T cells in the spleens of C57BL/6 wild-type (WT) mice that were treated with anti-PD-L1 or isotype on day −4, day −1, and day 2 and intravenously infected with 2 × 10^6^ PFU LCMV Docile on day 0 (*n* = 5; unpaired Student’s *t*-test). All data are shown as mean ± SEM. *p ≤ 0.05, **p ≤ 0.01, #*p* ≤ 0.001, and ##*p* ≤ 0.0001. In **(a–g)**, three independent experiments.

PD-L1 binds to its receptor, PD-1, and is found on activated T cells, B cells, and myeloid cells (32). PD-L1 interaction with PD-1 is essential for limiting T-cell activity and maintaining immune homeostasis, preventing immunopathology during chronic infection. To determine the consequences of impaired PD-L1 in aCDase-deficient mice, tamoxifen-treated CreER^+^ × *Asah1*^fl/fl^ and CreER^−^ × *Asah1*^fl/fl^ littermates were infected with 2 × 10^4^ PFU LCMV Docile and analyzed at 8 d.p.i. CreER^+^ × *Asah1*^fl/fl^ mice had significantly increased absolute numbers of splenic CD8^+^ T cells than CreER^−^ × *Asah1*^fl/fl^ controls, whereas CD4^+^ T-cell numbers were only modestly affected (**Figure 4a**). Baseline T-cell counts in uninfected, tamoxifen-treated mice were identical (**Figure 4b**), ruling out the intrinsic effects of *Asah1* deletion on T-cell development. Immunofluorescence confirmed heightened CD8^+^ T-cell infiltration in CreER^+^ spleens after infection (**Figure 4c**).

To investigate whether the increase in CD8^+^ T cells is virus-specific, we checked LCMV nucleoprotein (NP)-specific NP^+^CD8^+^ T-cell numbers and LCMV glycoprotein (GP33)-specific GP33^+^CD8^+^ T cells, which were significantly enhanced in CreER^+^ × *Asah1*^fl/fl^ compared to CreER^−^ × *Asah1*^fl/fl^ mice (**Figure 4d**). Functional capacity was tested by peptide restimulation: CD8^+^ T cells from CreER^+^ × *Asah1*^fl/fl^ mice produced significantly more IFN-γ than those from CreER^−^ × *Asah1*^fl/fl^ mice (**Figure 4f**; gating in **Figure 4e**), indicating enhanced effector function.

Finally, we asked whether loss of PD-L1 signaling alone could reproduce this phenotype. WT C57BL/6 mice treated with a blocking anti-PD-L1 antibody during LCMV infection exhibited a comparably strong CD8^+^ T-cell expansion (**Figure 4g**), confirming that insufficient PD-L1 signaling underlies the hyperactivation.

Collectively, these results show that aCDase deficiency limits PD-L1 upregulation, permitting uncontrolled expansion and effector activity of virus-specific CD8^+^ T cells during chronic LCMV infection.

### aCDase safeguards against lethal immunopathology during chronic LCMV infection

**Figure 5:**
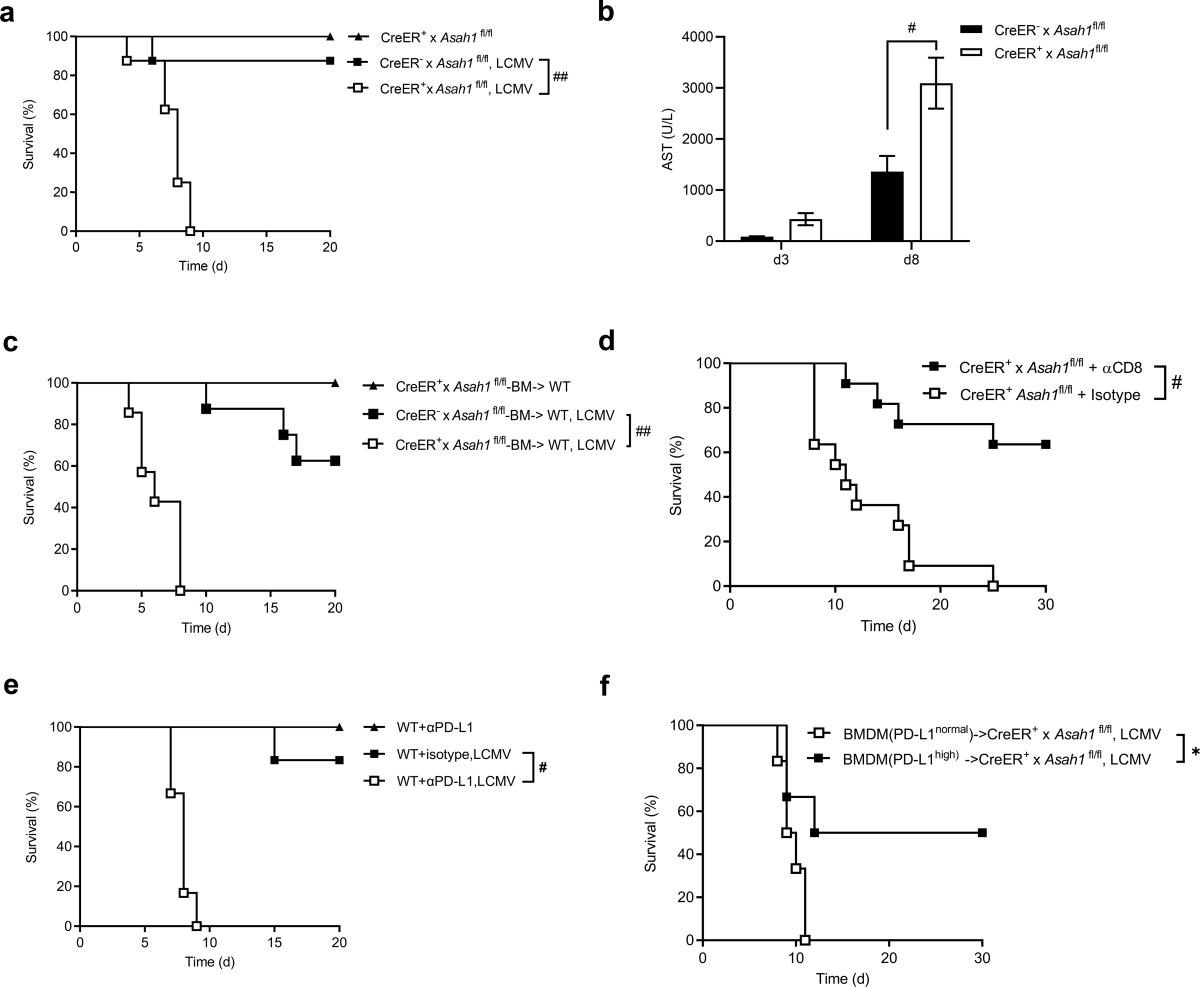
aCDase safeguards against lethal immunopathology during chronic LCMV infection. **(a)** Survival of tamoxifen-induced CreER^+^ × *Asah1*^fl/fl^ and CreER^−^ × *Asah1*^fl/fl^ mice infected with 2 × 10^6^ PFU LCMV Docile or without infection (naive; *n* = 8; log-rank [Mantel–Cox] test). **(b)** Levels of aspartate transaminase (AST) of serum on day 3 and day 8 from tamoxifen-induced CreER^+^ × *Asah1*^fl/fl^ and CreER^−^ × *Asah1*^fl/fl^ mice infected with 2 × 10^4^ PFU LCMV Docile on day 0 (*n* = 7–8; two-way ANOVA [Sidak’s multiple comparison]). **(c)** Survival of bone marrow chimeric mice receiving tamoxifen-treated CreER^+^ × *Asah1*^fl/fl^ or CreER^−^ × *Asah1*^fl/fl^ bone marrow and infected with 2 × 10^6^ PFU LCMV Docile (*n* = 7–8; log-rank [Mantel–Cox] test). **(d)** Survival of tamoxifen-treated CreER^+^ × *Asah1*^fl/fl^ mice that were treated with anti-CD8 or isotype (day −2, day 1, day 4) and intravenously infected with 8 × 10^4^ PFU LCMV Docile on day 0 (*n* = 11; log-rank [Mantel–Cox] test). **(e)** Survival of C57BL/6 wild-type (WT) mice that were treated with anti-PD-L1 or isotype on day −4, day −1, and day 2 and intravenously infected with 2 × 10^6^ PFU LCMV Docile on day 0 (*n* = 6; log-rank [Mantel–Cox] test). **(f)** Survival of tamoxifen-induced CreER^+^ × *Asah1*^fl/fl^ mice infected on day 1 with 2 × 10^6^ PFU LCMV Docile on day 0 and receiving 5 × 10^6^ bone marrow-derived macrophages (BMDMs) intravenously treated with PBS (BMDM+PD-L1^normal^) or IFN-α (BMDM+PD-L1^high^) 1 d.p.i. (*n* = 6; log-rank [Mantel–Cox] test). All data are shown as mean ± SEM. *p ≤ 0.05, #*p* ≤ 0.001, and ##*p* ≤ 0.0001. In **(a–f)**, two to three independent experiments.

To assess the *in-vivo* relevance of the ceramide → IFN-I → PD-L1 pathway, we infected tamoxifen-treated CreER^+^ × *Asah1*^fl/fl^ and CreER^−^ × *Asah1*^fl/fl^ littermates intravenously with LCMV Docile and monitored survival. CreER^+^ mice succumbed within 9 days, whereas CreER^−^ mice could control the infection and survived (**Figure 5a**). Consistent with immune-mediated tissue damage, CreER^+^ mice displayed marked liver injury, evidenced by elevated serum AST at day 8 d.p.i. (**Figure 5b**). To confirm whether aCDase expression in the hematopoietic compartment was sufficient for protection, we generated bone marrow (BM) chimeras by transplanting CreER^+^ × *Asah1*^fl/fl^ or CreER^−^ × *Asah1*^fl/fl^ BM into irradiated C57BL/6 WT mice recipients. After tamoxifen induction, mice with aCDase-deficient immune cells died significantly earlier than WT control BM (**Figure 5c**), indicating an immune cell-intrinsic requirement for aCDase to protect against lethal LCMV Docile infection.

WT mice efficiently clear the acute LCMV-WE strain by day 8, whereas tamoxifen-treated CreER^+^ × *Asah1*^fl/fl^ animals fail to do so: virus titers remain high in every organ examined, and the mice succumb rapidly (**Supplementary Figures 4a, b**). A similar viral persistence is seen in *Ifnar*^−/−^ mice, yet they survive because LCMV-WE is non-cytopathic. By contrast, during infection with the chronic LCMV Docile strain, early viral loads are comparable in wild-type and CreER^+^ animals (**Figure 1h**), but only the aCDase-deficient mice die (**Figure 5c**). These observations indicate that lethality arises from immunopathology driven by an overactive CD8^+^ T-cell response, rather than from uncontrolled virus replication.

To test whether hyperactive CD8^+^ T cells drive this pathology, we depleted CD8^+^ T cells in CreER^+^ × *Asah1*^fl/fl^ mice. CD8^+^ cell depletion significantly prolonged survival (**Figure 5d**). Conversely, T-cell-specific *Asah1* deletion (CD4^+^Cre × *Asah1*^fl/fl^) did not reproduce the phenotype (**Supplementary Figure 5**), showing that aCDase acts upstream of T cells, consistent with its role in pDCs.

To examine whether insufficient PD-L1 signaling drives pathology, we blocked PD-L1 in WT mice during infection. Anti-PD-L1 treatment reduced survival to a degree similar to CreER^+^ × *Asah1*^fl/fl^ mice (**Figure 5e**). Conversely, adoptive transfer of bone marrow-derived macrophages pretreated with IFN-α to induce high PD-L1 (PD-L1^high^ BMDMs) significantly improved the survival of infected CreER^+^ × *Asah1*^fl/fl^ mice, whereas PD-L1^normal^ BMDMs did not (**Figure 5f**).

Taken together, these data demonstrate that aCDase-driven ceramide homeostasis prevents fatal immunopathology during chronic LCMV infection by enabling IFN-I-dependent PD-L1 expression and the timely exhaustion of virus-specific CD8^+^ T cells. Loss of aCDase in immune cells disrupts this checkpoint, unleashing cytotoxic T-cell-mediated tissue damage.

## Discussion

Viruses thrive or fail according to how effectively the host calibrates type I interferon signaling. Here, we reveal that this calibration is not controlled solely by canonical pattern recognition pathways, but by a lipid-metabolic “rheostat”: acid ceramidase (aCDase). By limiting ceramide accumulation in endolysosomal membranes, aCDase licenses a vigorous early IFN-I burst from pDCs, triggers timely PD-L1 upregulation on antigen-presenting cells (APCs), and thereby enforces protective CD8^+^ T-cell exhaustion. Loss of aCDase disrupts this cascade and converts a normally non-lethal chronic LCMV infection into fatal immunopathology.

Beyond serving as static membrane building blocks, sphingolipids orchestrate diverse cellular programs ranging from survival and proliferation (24, 33) to tumor immunology and therapy (34, 35). Dysregulation of sphingolipid metabolism has also been implicated in the pathogenesis of several infectious diseases, including HSV-1 encephalitis, COVID-19 (SARS-CoV-2), measles, and bacterial pneumonia (25, 36–39). Hydrolyzing ceramide to sphingosine and fatty acid, aCDase sits at the fulcrum of this lipid network, yet its immunological role, especially in professional interferon producers, has remained obscure. Our findings position aCDase-controlled ceramide balance as a master switch that calibrates endosomal nucleic acid signaling and thereby sets the amplitude of the innate antiviral response.

A key mechanistic insight from our study is that ceramide abundance alters the biophysical integrity of endosomal signaling microdomains. Excess ceramide in aCDase-deficient pDCs likely disrupts these domains by increasing negative membrane curvature, dispersing TLR nanoclusters, delaying IRF5/7 phosphorylation, and sharply attenuating IFN-α/β output. In contrast, reducing ceramide—for example, via acid sphingomyelinase (ASM) deficiency, stabilizes these microdomains and enhances IFN-I responses, establishing a bidirectional “rheostat” model. Importantly, the effect extends beyond a single receptor; multiple TLR pathways (TLR9, TLR7/8, and TLR3) are impacted, highlighting a broad influence of ceramide-regulated lipid architecture rather than individual receptors. Future studies employing super-resolution microscopy and targeted lipidomics will be essential to validate this model experimentally and to provide detailed insights into the exact ceramide subspecies and related lipid metabolites involved.

The PD-L1/PD-1 checkpoint is essential for limiting tissue damage in persistent infections. Our study links sphingolipid homeostasis directly to this checkpoint: when aCDase is absent, ceramide accumulates, IFN-I is blunted, and PD-L1 induction fails, allowing unchecked expansion of virus-specific CD8^+^ T cells. This aligns with earlier work showing that IFN-I licenses PD-L1 to prevent pathology in chronic LCMV (40). Therapeutically, manipulating ceramide, via aCDase activation or ASM inhibition, could either bolster early interferon and PD-L1 induction (to protect tissues) or, conversely, transiently reduce PD-L1 to improve vaccine responses, provided safety can be ensured. Nanoparticle-based delivery systems already under clinical evaluation could be adapted to deliver lipid-modifying agents directly to pDCs (41). These findings offer the first molecular link between ceramide-regulated membrane microdomains and the PD-L1 checkpoint governing CD8^+^ T-cell exhaustion.

From a translational angle, our data position aCDase as a druggable “gain knob” for modulating IFN-I and PD-L1 responses. Recombinant aCDase has already been shown to normalize ceramide levels and is well-tolerated in models of Farber disease (42). Clinically approved functional inhibitors of acid sphingomyelinase—such as the licensed antidepressant amitriptyline—lower ceramide and block SARS-CoV-2 entry into primary human airway epithelia (43), illustrating pathway tractability in humans. Additionally, potent first-generation small molecule aCDase inhibitors with submicromolar cellular activity have been reported (44), providing starting points for drug discovery aimed at fine-tuning checkpoint-ligand expression in chronic viral hepatitis or HIV. Nevertheless, side effects from systemic ceramide modulation, as well as tissue-specific targeting barriers, will need careful evaluation during therapeutic development.

Advanced single-cell and next-generation spatial technologies will be invaluable to pinpoint the precise IFN-I producer–responder circuits regulated by the ceramide rheostat. Approaches such as Perturb-DBiT spatial CRISPR screening (45); spatial tri-omics mapping integrating chromatin, RNA, and protein (46); and high-plex protein plus transcript co-mapping (47) now enable resolution of pDC, monocyte, and macrophage niches at subcellular precision. Our IFN-β stimulation experiments already reveal clear responder heterogeneity, with dendritic cells uniquely sensitive to ceramide modulation. Moreover, the impaired PD-L1 induction observed in aCDase-deficient BMDCs suggests an additional intrinsic role for altered ceramide distribution in directly modulating PD-L1 surface expression, beyond simply impairing IFN-I responsiveness. Comprehensive single-cell and spatial transcriptomic analyses in future studies will thus be essential for definitively delineating the ceramide-regulated IFN-I producer–responder network, clarifying both IFN-dependent and intrinsic ceramide-mediated effects.

Several questions remain. At the transcriptional level, it will be important to test whether ceramide also modulates PD-L1 via IRF4 or BATF, two lipid-sensitive transcription factors previously implicated in PD-L1 upregulation (48). High-resolution lipidomics in patient pDCs and spatial imaging of ceramide microdomains will be the crucial next steps. Intriguingly, the Docile strain replicates slowly to minimize early interferon induction (49); whether manipulation of host aCDase or ASM contributes to this strategy remains to be determined.

Our study has several limitations. All data were generated in mice, so translating the findings from LCMV to chronic human infections, such as HBV, HIV, or CMV, will require careful validation, given species-specific differences in immune exhaustion and sphingolipid metabolism. Lethality in *Asah1*-deficient animals after days 7–9 prevented us from sampling later time points and thus from following IFN-I/PD-L1 dynamics into true chronicity. Additionally, we used a global, inducible knockout model; thus, indirect effects from other APCs cannot be excluded. Although our findings implicate pDCs as the principal effectors, only pDC-specific deletion or targeted depletion will definitively establish cellular specificity. Achieving such lineage-specific manipulation in a rare population remains technically demanding but is essential. Finally, the data suggest that the aCDase–ceramide axis acts as a lipid-metabolic “gain knob,” modulating endosomal microdomain integrity upstream of classical IFN-I regulators (TLRs, MyD88, IRF3/7). Future work using conditional knockouts, APC subset profiling, and humanized models will be critical to refine mechanistic attribution and translation to therapeutic settings.

## Conclusion

We identify aCDase-regulated ceramide balance as a master switch that calibrates endosomal nucleic acid sensing, IFN-I amplitude, and PD-L1-dependent CD8^+^ T-cell exhaustion. Targeting this lipid “gain knob” may offer a novel therapeutic axis to balance antiviral immunity and immunopathology in chronic viral infections. Future development of small molecule aCDase activators or ASM inhibitors, combined with testing in preclinical models of hepatitis or respiratory viruses, could pave the way for clinically viable strategies that preserve host defense while limiting immune-mediated tissue damage.

## Materials and methods

### Mice

Acid ceramidase-deficient mice CreER × *Asah1*^fl/fl^ and *Asah1*^−/−^ (*Asah1*^fl/fl^ × EIIa-Cre) mice were created as previously described (25). Tamoxifen was dissolved in corn oil. Eight, 6, and 4 days before an experiment, Cre^−^ × *Asah1*^fl/f^*^l^* control animals and Cre^+^ × *Asah1*^fl/fl^ animals were treated with 4 mg of tamoxifen (in 100 μL of corn oil) intraperitoneally. IFNAR^−/−^ mice were generated as described previously (50).

All animals were housed in single ventilated cages. Animal experiments were authorized by the Landesamt für Natur, Umwelt und Verbraucherschutz (LANUV) Nordrhein-Westfalen and in accordance with the German law for animal protection and/or according to institutional guidelines at the Ontario Cancer Institute of the University Health Network and/or according to the animal care and use committee of Peking University Cancer Hospital (ZY202402).

#### Anesthesia and euthanasia

Retro-orbital blood sampling was performed under brief isoflurane anesthesia delivered via a precision vaporizer (induction 5 vol%, maintenance 2.5–3 vol% in O_2_). For terminal procedures, mice were euthanized by cervical dislocation without prior anesthesia, followed immediately by cardiac puncture for blood collection and/or organ harvest.

### Virus

The LCMV Docile strain was originally obtained from Dr. C. J. Pfau (Troy, New York). Viruses were propagated in L929 cells (obtained from the ATCC; NCTC clone 929). Mice were intravenously challenged with 2 × 10^6^ PFU LCMV *in-vivo* experiment if not mentioned otherwise and with MOI 1 for *in-vitro* experiments.

### Reagents and antibodies

For flow cytometry and immunofluorescence assays, we used the following antibodies specific for given antigens: anti-CD274 (PD-L1) (Cat # 12-5982-82, eBioscience, San Diego, CA, USA, 1:100 for FACS, 1:200 for histology), anti-Ly6C (Cat # 17-5932-82, eBioscience, San Diego, CA, USA, 1:100), anti-CD11b (Cat # 11-0112-41, eBioscience, San Diego, CA, USA, 1:100), anti-CD3e (Cat # 11-0031-82, eBioscience, San Diego, CA, USA, 1:100), anti-CD4 (Cat # 17-0042-83, eBioscience, San Diego, CA, USA, 1:100), anti-CD8a (Cat # 12-0081-85, eBioscience, San Diego, CA, USA, 1:100), anti-IFNγ (Cat # 17-7311-82, eBioscience, San Diego, CA, USA, 1:100), and anti-CD8a (Cat # 500081-82, eBioscience, San Diego, CA, USA, 1:100).

Reagents used for other analyses were Recombinant Mouse IFN-beta Protein (Biotech, 8234-MB-010) and Recombinant Mouse IFN-alpha Protein (Biotech, 12100-1). For *in-vivo* treatment, we used InVivoMAb anti-mouse PD-L1 (B7-H1) (BioXcell, Lebanon, NH, USA, #BE0101), InVivoMAb anti-mouse CD8α (BioXcell, Lebanon, NH, USA), or Rat IgG2b isotype control (BioXcell, Lebanon, NH, USA, #BE0090).

### Microarray analysis

The GEO repository series GSE75202 was downloaded to analyze the *Asah1* expression in 11 different immunocyte populations in mice (core ImmGen first-generation 11-cell set). The relative expression was normalized by GAPDH. A list of all GEO accession numbers and corresponding cell populations and series is available as **Supplementary Table S1** in the Supplementary Material.

### Generation and *in-vitro* stimulation of mouse BMDMs, BMDCs, and Flt3L-DCs

For bone marrow-derived macrophage (BMDM) generation, bone marrow was isolated from the legs of mice and cultured for 9 days with 20 ng/mL of macrophage colony-stimulating factor (M-CSF) (PeproTech, Rocky Hill, NJ, USA), and 50% of the culture medium was replenished after 5 days. For GM-CSF bone marrow dendritic cell (BMDC) generation, bone marrow cells were cultured in the presence of 20 ng/mL of murine granulocyte-macrophage colony-stimulating factor (GM-CSF) (PeproTech, Rocky Hill, NJ, USA) and 4 ng/mL of IL-4 (PeproTech, Rocky Hill, NJ, USA) for 6–7 days, and 50% of the culture medium was replenished after 3 days. Flt3L-DCs were generated by BM cells in RPMI medium 1640 (Gibco, Waltham, MA, USA), supplemented with 100 ng/mL of murine Fms-like tyrosine kinase 3 ligand (rFlt-3L; R&D Systems, Minneapolis, MN, USA) for 7 days, and 50% of the culture medium was replenished after 5 days. A total of 1 million/mL cells were plated on 24-well plates, and the experiment was started the next day.

### Plaque-forming assay

We utilized the plaque-forming assay and MC57 fibroblasts as previously described (51). Briefly, the organs smashed in Dulbecco’s modified Eagle medium (DMEM) supplemented with 2% fetal calf serum (FCS) were titrated 1:3 over 12 steps and plaqued onto MC57 fibroblasts. After 3 h, methylcellulose 1% medium was added. After 2 days of incubation, viruses were visualized by staining against LCMV nucleoprotein via an anti-LCMV-NP antibody (clone VL-4).

### Interferon measurement

Serum from mice was isolated at 0.5-, 1-, 2-, and 3 days post-infection (d.p.i.). Flt3L-DCs were seeded at a cell density of 1 × 10^6^ cells/mL, followed by adding 1 μM of Dotap-CpG overnight. For maximum IFN-α/β response, 1 μM of CpG was complexed with Dotap (Roche, Basel, Switzerland). In detail, 10 nmol of CpG was diluted in 100 µL of HBSS and 30 µL of Dotap was diluted in another 70 µL of HBSS. The diluted CpG and Dotap were mixed and incubated at room temperature for 15 min and then diluted with 10 mL of medium to make 1 µM of Dotap-CpG (52). IFN-α and IFN-β in Flt3L-DC supernatants and mice sera were measured by the LEGENDplex™ Anti-Virus Response Panel according to the manufacturer’s protocol (BioLegend, San Diego, CA, USA). The concentration of a particular analyte was determined based on a known standard curve using the LEGENDplex™ data analysis software.

### Immunofluorescence microscopy

Flt3L-DCs were seeded on coverslips (covered by collagen) and untreated or treated with 1 µM of DOTAP-CpG for 3 h. The cells were fixed and permeabilized. The following primary antibodies were used: anti-ceramide (Glycobiotech GmbH, Kükels, Germany, MAB_0011, 1:100), anti-Rab5 (Cell Signaling, Danvers, MA, USA, 3547s, 1:200), and FITC anti-TLR9 antibody (Abcam, Cambridge, UK, ab210925). Then, staining with the respective Cy3-conjugated donkey anti-mouse IgM (AB_2340815, 1:200) and FITC-conjugated goat anti-rabbit IgG (111-095-144, 1:200) was conducted. The nuclei were stained with 4′,6-diamidino-2-phenylindole (DAPI). A Leica SP8 gSTED and FLIM microscope was used to acquire images.

### Cytokine measurement

R848 (InvivoGen), ODN 1585-TLR9 ligand class A (CpG, InvivoGen), and polyinosinic:polycytidylic acid (poly(I:C), Sigma, Darmstadt, Germany) were added as indicated. IL-6, TNF-α, IL-10, IL-1β, and IL-12p70 in BMDC supernatant following stimulation with CpG, R848+poly (I:C) were measured after an overnight incubation or as indicated. All samples were measured by the LEGENDplex™ Anti-Virus Response Panel according to the manufacturer’s protocol (BioLegend, San Diego, CA, USA). The concentration of a particular analyte was determined based on a known standard curve using the LEGENDplex™ data analysis software.

### Quantitative real-time polymerase chain reaction

BMDCs and BMDMs were stimulated for 24 h and then subjected to quantitative real-time PCR analysis. Mice spleens were isolated on day 3 after virus infection. Total RNA was extracted with TRIzol (Life Technologies, Carlsbad, CA, USA). The RNA was reverse-transcribed into cDNA with the Quantitect Reverse Transcription Kit (Qiagen, Hilden, Germany). Gene expression analysis was performed with assays from Qiagen: glyceraldehyde 3-phosphate dehydrogenase (GAPDH; QT01658692); IFN-α fwd (seq: 5′-ATG GCT AGR CTC TCT GCT TTC CT-3′), IFN-α rev (seq: 5′-AGG GCT CTC CAG AYT TCT GCT CTG-3′); IFN-β fwd (seq: 5′-CAG GCA ACC TTT AAG CAT CAG-3′), IFN-β rev (seq: 5′-CCT TTG ACC TTT CAA ATG CAG-3′); and Pdcd1lg1(CD274-PD-L1, QT00148617). Data were normalized to the level of GAPDH expression in each sample. Relative quantities (RQs) were determined with the equation: RQ = 2^−ddCt^.

### Flow cytometry

Experiments were performed using fluorescence‐activated cell sorting (BD LSRFortessa™ cell analyzer) and analyzed using FlowJo V10 software. For cell subset and surface molecule staining, single suspended cells were incubated with surface antibodies for 30 min at 4°C.

### Tetramer staining

Tetramers were provided by the National Institutes of Health (NIH) Tetramer Facility (Emory University, Atlanta, GA, USA). Cells were stained with allophycocyanin (APC)-labeled NP or GP33 major histocompatibility complex class I tetramers for 15 min at 37°C. After incubation, the samples were stained with anti-CD8 (BD Biosciences, San Diego, NJ, USA) for 30 min at 4°C. Erythrocytes were then lysed with BD lysing solution (BD Biosciences), washed once, and analyzed by flow cytometry. Absolute numbers of NP-specific CD8^+^ T cells or GP33-specific CD8^+^ T cells per microliter of spleen were determined by fluorescence-activated cell sorting (FACS) analysis using fluorescent beads (BD Biosciences). All antibodies were diluted 1:100 to their original concentration in FACS buffer. For the determination of total cell numbers, FACS beads were used (BD Biosciences). All stained cells were analyzed on an LSR II or a FACS Fortessa flow cytometer (BD Biosciences), and data were analyzed with the FlowJo software (FlowJo LLC, Ashland, OR, USA).

### Intracellular cytokine staining

Splenic tissue was homogenized, and splenocytes were restimulated with LCMV-GP33-specific peptide (PolyPeptide Laboratories, Strasbourg, France); after 2 h, brefeldin A (BFA) was added and incubated at 37°C for 6 h. Then, it was washed twice before surface antibodies and intracellular markers were added. In short, anti-CD8 were used for surface staining. For measurement of intracellular IFN-γ, cells were fixed with formaldehyde (2% formaldehyde solution in PBS) for 10 min, permeabilized with saponin (1%) solution, and stained with anti-IFN-γ antibodies.

### Histology

Histologic analyses used snap-frozen tissue. Sections were stained with anti-PD-L1 and anti-CD8. In short, sections were fixed with acetone for 10 min, air-dried for 10 min, and blocked in 2% FCS-PBS for 15 min, followed by staining with various antibodies for 45 min. All antibodies were diluted 1:100 from their original concentration in blocking solution. Images of the stained sections were acquired with a fluorescence microscope (KEYENCE BZ II analyzer; KEYENCE Corporation of America, Itasca, IL, USA).

### Bone marrow chimeras

C57BL/6 (WT) mice (recipient) were irradiated with 9.5 Gy. The following day, irradiated mice were reconstituted by intravenous injection of 5 × 10^6^ bone marrow cells from donor mice (CreER^+^ × *Asah1*^fl/fl^ and CreER^−^ × *Asah1*^fl/fl^) to the recipient. On day 10 after irradiation, mice were treated with clodronate liposomes to deplete tissue-resident macrophages. After 50 days of reconstitution, tamoxifen was administered to mice every other day three times. Mice were ready to be used in experiments.

### Western blot

Cells were lysed in radioimmunoprecipitation buffer containing 1× HALT Protease Inhibitor Cocktail and 50 mM of EDTA (Thermo Fisher, Waltham, MA, USA, Cat. no. 78430). Protein concentration was measured using detergent-compatible (DC) Protein Assay (Bio-Rad, Hercules, CA, USA, Cat. no. 5000112). A total of 30 µg per cell lysate was loaded per lane into a precast TGX AnyKD Stain-free gel (Bio-Rad, Hercules, CA, USA, Cat. no. 4568126). Transfer was performed using the Trans-Blot Turbo system (Bio-Rad, Hercules, CA, USA, Cat. no. 1704150) with Mini PVDF Transfer Packs (Bio-Rad, Hercules, CA, USA, Cat. no. 1704156). Anti-phospho-IRF5 rabbit (Thermo Fisher, Waltham, MA, USA, PA5-64760), anti-phospho-IRF7 rabbit (Thermo Fisher, Waltham, MA, USA, PA5-114592), and anti-β-actin mouse (Sigma, Darmstadt, Germany, A2228) were used for primary antibody incubation overnight. Then, membranes were washed three times with Tris-buffered saline-0.1% Tween 20 (TBS-T) and incubated with secondary antibody HRP-linked anti-rabbit IgG or HRP-linked anti-mouse IgG (Cell Signaling, Danvers, MA, USA). After washing three times in TBS-T, membranes were incubated with Pierce ECL (Thermo Fisher, Waltham, MA, USA, Cat. no. 32106) for 1 min and imaged on a Bio-Rad ChemiDoc MP (Cat. no. 1708280) with ImageLab version 6.0.1 (Bio-Rad, Hercules, CA, USA).

### Survival experiments

Animals were intravenously (i.v.) challenged with 2 × 10^6^ PFU LCMV for the survival experiments. A total of 250 µg/mouse of anti-PD-L1 antibody (BioXcell, Lebanon, NH, USA) was injected intraperitoneally on day −4, day −1, and day 2 of virus infection. For the CD8^+^ T-cell depletion experiment, mice were injected with 8 × 10^4^ PFU LCMV. A total of 100 µg/mouse of anti-CD8 (BioXcell, Lebanon, NH, USA) or isotype (BioXcell, Lebanon, NH, USA) was injected intraperitoneally on day −2, day 1, and day 4 of virus infection.

Animals were checked daily, killed with corresponding termination criteria, and counted as dead. Termination criteria were body weight, general condition, spontaneous behavior, and clinical findings.

### Statistical analysis

All data are shown as mean ± SEM. The level of statistical significance was set at *p ≤ 0.05, **p ≤ 0.01, ^#^*p* ≤ 0.001, and ^##^*p* ≤ 0.0001.

## Data Availability

The original contributions presented in the study are included in the article/**Supplementary Material**. Further inquiries can be directed to the corresponding authors.

## References

[1] Muller U Steinhoff U Reis LF Hemmi S Pavlovic J Zinkernagel RM . Functional role of type I and type II interferons in antiviral defense. Science. (1994) 264:1918–21. doi: 10.1126/science.8009221, PMID: 8009221

[2] Schneider WM Chevillotte MD Rice CM . Interferon-stimulated genes: a complex web of host defenses. Annu Rev Immunol. (2014) 32:513–45. doi: 10.1146/annurev-immunol-032713-120231, PMID: 24555472 PMC4313732

[3] Clemens MJ Williams BR . Inhibition of cell-free protein synthesis by pppA2’p5’A2’p5’A: a novel oligonucleotide synthesized by interferon-treated L cell extracts. Cell. (1978) 13:565–72. doi: 10.1016/0092-8674(78)90329-x, PMID: 657268

[4] Haller O Arnheiter H Lindenmann J Gresser I . Host gene influences sensitivity to interferon action selectively for influenza virus. Nature. (1980) 283:660–2. doi: 10.1038/283660a0, PMID: 7354853

[5] Roberts WK Hovanessian A Brown RE Clemens MJ Kerr IM . Interferon-mediated protein kinase and low-molecular-weight inhibitor of protein synthesis. Nature. (1976) 264:477–80. doi: 10.1038/264477a0, PMID: 1004583

[6] Swiecki M Omattage NS Brett TJ . BST-2/tetherin: structural biology, viral antagonism, and immunobiology of a potent host antiviral factor. Mol Immunol. (2013) 54:132–9. doi: 10.1016/j.molimm.2012.11.008, PMID: 23274150

[7] Shaabani N Duhan V Khairnar V Gassa A Ferrer-Tur R Häussinger D . CD169(+) macrophages regulate PD-L1 expression via type I interferon and thereby prevent severe immunopathology after LCMV infection. Cell Death Dis. (2016) 7:e2446. doi: 10.1038/cddis.2016.350, PMID: 27809306 PMC5260878

[8] Marshall JS Warrington R Watson W Kim HL . An introduction to immunology and immunopathology. Allergy Asthma Clin Immunol. (2018) 14:49. doi: 10.1186/s13223-018-0278-1, PMID: 30263032 PMC6156898

[9] Nirmal AJ Maliga Z Vallius T Quattrochi B Chen AA Jacobson CA . The spatial landscape of progression and immunoediting in primary melanoma at single-cell resolution. Cancer Discov. (2022) 12:1518–41. doi: 10.1158/2159-8290.Cd-21-1357, PMID: 35404441 PMC9167783

[10] Teijaro JR Ng C Lee AM Sullivan BM Sheehan KC Welch M . Persistent LCMV infection is controlled by blockade of type I interferon signaling. Science. (2013) 340:207–11. doi: 10.1126/science.1235214, PMID: 23580529 PMC3640797

[11] Wherry EJ . T cell exhaustion. Nat Immunol. (2011) 12:492–9. doi: 10.1038/ni.2035, PMID: 21739672

[12] Barchet W Cella M Odermatt B Asselin-Paturel C Colonna M Kalinke U . Virus-induced interferon alpha production by a dendritic cell subset in the absence of feedback signaling *in vivo*. J Exp Med. (2002) 195:507–16. doi: 10.1084/jem.20011666, PMID: 11854363 PMC2193622

[13] Fitzgerald-Bocarsly P Dai J Singh S . Plasmacytoid dendritic cells and type I IFN: 50 years of convergent history. Cytokine Growth Factor Rev. (2008) 19:3–19. doi: 10.1016/j.cytogfr.2007.10.006, PMID: 18248767 PMC2277216

[14] Huang X Yang Y . Targeting the TLR9-MyD88 pathway in the regulation of adaptive immune responses. Expert Opin Ther Targets. (2010) 14:787–96. doi: 10.1517/14728222.2010.501333, PMID: 20560798 PMC2917181

[15] Benmohamed F Medina M Wu YZ Maschalidi S Jouvion G Guillemot L . Toll-like receptor 9 deficiency protects mice against Pseudomonas aeruginosa lung infection. PloS One. (2014) 9:e90466. doi: 10.1371/journal.pone.0090466, PMID: 24595157 PMC3942450

[16] Stith JL Velazquez FN Obeid LM . Advances in determining signaling mechanisms of ceramide and role in disease. J Lipid Res. (2019) 60:913–8. doi: 10.1194/jlr.S092874, PMID: 30846529 PMC6495170

[17] Grassmé H Schwarz H Gulbins E . Molecular mechanisms of ceramide-mediated CD95 clustering. Biochem Biophys Res Commun. (2001) 284:1016–30. doi: 10.1006/bbrc.2001.5045, PMID: 11409897

[18] Hailemariam TK Huan C Liu J Li Z Roman C Kalbfeisch M . Sphingomyelin synthase 2 deficiency attenuates NFkappaB activation. Arterioscler Thromb Vasc Biol. (2008) 28:1519–26. doi: 10.1161/atvbaha.108.168682, PMID: 18566297

[19] Bezgovsek J Gulbins E Friedrich SK Lang KS Duhan V . Sphingolipids in early viral replication and innate immune activation. Biol Chem. (2018) 399:1115–23. doi: 10.1515/hsz-2018-0181, PMID: 29975662

[20] Schuchman EH . Acid ceramidase and the treatment of ceramide diseases: The expanding role of enzyme replacement therapy. Biochim Biophys Acta. (2016) 1862:1459–71. doi: 10.1016/j.bbadis.2016.05.001, PMID: 27155573

[21] Tsuboi K Tai T Yamashita R Ali H Watanabe T Uyama T . Involvement of acid ceramidase in the degradation of bioactive N-acylethanolamines. Biochim Biophys Acta Mol Cell Biol Lipids. (2021) 1866:158972. doi: 10.1016/j.bbalip.2021.158972, PMID: 34033896

[22] Utermöhlen O Herz J Schramm M Krönke M . Fusogenicity of membranes: the impact of acid sphingomyelinase on innate immune responses. Immunobiology. (2008) 213:307–14. doi: 10.1016/j.imbio.2007.10.016, PMID: 18406376

[23] Abe A Shayman JA . Sphingolipid catabolism. In: Lennarz WJ Lane MD , editors. Encyclopedia of biological chemistry, 2nd ed. Waltham, MA, USA: Academic Press (2013). p. 287–92. doi: 10.1016/B978-0-12-378630-2.00462-X

[24] Beck M Moser HW Sandhoff K . Chapter 39 - Acid ceramidase deficiency: Farber lipogranulomatosis, spinal muscular atrophy associated with progressive myoclonic epilepsy and peripheral osteolysis. In: Rosenberg RN Pascual JM , editors. Rosenberg’s molecular and genetic basis of neurological and psychiatric disease, Sixth Edition. Cambridge, MA, USA and London, UK: Academic Press (2020). p. 547–57. doi: 10.1016/B978-0-12-813955-4.00039-8

[25] Lang J Bohn P Bhat H Jastrow H Walkenfort B Cansiz F . Acid ceramidase of macrophages traps herpes simplex virus in multivesicular bodies and protects from severe disease. Nat Commun. (2020) 11:1338. doi: 10.1038/s41467-020-15072-8, PMID: 32165633 PMC7067866

[26] Lutz MB Ali S Audiger C Autenrieth SE Berod L Bigley V . Guidelines for mouse and human DC generation. Eur J Immunol. (2022) 53(11):e2249816. doi: 10.1002/eji.202249816, PMID: 36303448 PMC10704271

[27] Webster B Assil S Dreux M . Cell-cell sensing of viral infection by plasmacytoid dendritic cells. J Virol. (2016) 90:10050–3. doi: 10.1128/jvi.01692-16, PMID: 27605675 PMC5105643

[28] Ito T Wang YH Liu YJ . Plasmacytoid dendritic cell precursors/type I interferon-producing cells sense viral infection by Toll-like receptor (TLR) 7 and TLR9. Springer Semin Immunopathol. (2005) 26:221–9. doi: 10.1007/s00281-004-0180-4, PMID: 15592841

[29] Henry B Ziobro R Becker KA Kolesnick R Gulbins E . Acid sphingomyelinase. Handb Exp Pharmacol. (2013) 215:77–88. doi: 10.1007/978-3-7091-1368-4_4, PMID: 23579450

[30] Liu J Zhang E Ma Z Wu W Kosinska A Zhang X . Enhancing virus-specific immunity *in vivo* by combining therapeutic vaccination and PD-L1 blockade in chronic hepadnaviral infection. PloS Pathog. (2014) 10:e1003856. doi: 10.1371/journal.ppat.1003856, PMID: 24391505 PMC3879364

[31] Yamazaki T Akiba H Iwai H Matsuda H Aoki M Tanno Y . Expression of programmed death 1 ligands by murine T cells and APC. J Immunol. (2002) 169:5538–45. doi: 10.4049/jimmunol.169.10.5538, PMID: 12421930

[32] Blackburn SD Crawford A Shin H Polley A Freeman GJ Wherry EJ . Tissue-specific differences in PD-1 and PD-L1 expression during chronic viral infection: implications for CD8 T-cell exhaustion. J Virol. (2010) 84:2078–89. doi: 10.1128/jvi.01579-09, PMID: 19955307 PMC2812396

[33] Park JH Schuchman EH . Acid ceramidase and human disease. Biochim Biophys Acta. (2006) 1758:2133–8. doi: 10.1016/j.bbamem.2006.08.019, PMID: 17064658

[34] Lai M La Rocca V Amato R Freer G Costa M Spezia PG . Ablation of acid ceramidase impairs autophagy and mitochondria activity in melanoma cells. Int J Mol Sci. (2021) 22:3247. doi: 10.3390/ijms22063247, PMID: 33806766 PMC8004726

[35] Liu X Elojeimy S Turner LS Mahdy AE Zeidan YH Bielawska A . Acid ceramidase inhibition: a novel target for cancer therapy. Front Biosci. (2008) 13:2293–8. doi: 10.2741/2843, PMID: 17981711

[36] Becker KA Verhaegh R Verhasselt HL Keitsch S Soddemann M Wilker B . Acid ceramidase rescues cystic fibrosis mice from pulmonary infections. Infect Immun. (2021) 89:e00677-20. doi: 10.1128/iai.00677-20, PMID: 33139382 PMC7822142

[37] Geiger N Kersting L Schlegel J Stelz L Fähr S Diesendorf V . The acid ceramidase is a SARS-coV-2 host factor. Cells. (2022) 11(16):2532. doi: 10.3390/cells11162532, PMID: 36010608 PMC9406565

[38] Grafen A Schumacher F Chithelen J Kleuser B Beyersdorf N Schneider-Schaulies J . Use of acid ceramidase and sphingosine kinase inhibitors as antiviral compounds against measles virus infection of lymphocytes *in vitro*. Front Cell Dev Biol. (2019) 7:218. doi: 10.3389/fcell.2019.00218, PMID: 31632969 PMC6779704

[39] Kornhuber J Hoertel N Gulbins E . The acid sphingomyelinase/ceramide system in COVID-19. Mol Psychiatry. (2022) 27:307–14. doi: 10.1038/s41380-021-01309-5, PMID: 34608263 PMC8488928

[40] Wilson EB Yamada DH Elsaesser H Herskovitz J Deng J Cheng G . Blockade of chronic type I interferon signaling to control persistent LCMV infection. science. (2013) 340:202–7. doi: 10.1126/science.1235208, PMID: 23580528 PMC3704950

[41] Liu P Chen G Zhang J . A review of liposomes as a drug delivery system: current status of approved products, regulatory environments, and future perspectives. Molecules. (2022) 27:1372. doi: 10.3390/molecules27041372, PMID: 35209162 PMC8879473

[42] He X Dworski S Zhu C DeAngelis V Solyom A Medin JA . Enzyme replacement therapy for Farber disease: Proof-of-concept studies in cells and mice. BBA Clin. (2017) 7:85–96. doi: 10.1016/j.bbacli.2017.02.001, PMID: 28275553 PMC5338723

[43] Carpinteiro A Edwards MJ Hoffmann M Kochs G Gripp B Weigang S . Pharmacological inhibition of acid sphingomyelinase prevents uptake of SARS-coV-2 by epithelial cells. Cell Rep Med. (2020) 1:100142. doi: 10.1016/j.xcrm.2020.100142, PMID: 33163980 PMC7598530

[44] Realini N Solorzano C Pagliuca C Pizzirani D Armirotti A Luciani R . Discovery of highly potent acid ceramidase inhibitors with *in vitro* tumor chemosensitizing activity. Sci Rep. (2013) 3:1035. doi: 10.1038/srep01035, PMID: 23301156 PMC3539145

[45] Fan R Baysoy A Tian X Zhang F Renauer P Bai Z . Spatially Resolved Panoramic *in vivo* CRISPR Screen via Perturb-DBiT. Res Sq. (2025). doi: 10.21203/rs.3.rs-6481967/v1, PMID: 40386382 PMC12083649

[46] Fan R Zhang D Rodriguez-Kirby L Lin Y Song M Wang L . Spatial dynamics of mammalian brain development and neuroinflammation by multimodal tri-omics mapping. Res square. (2024). doi: 10.21203/rs.3.rs-4814866/v1, PMID: 39184075 PMC11343178

[47] Liu Y DiStasio M Su G Asashima H Enninful A Qin X . High-plex protein and whole transcriptome co-mapping at cellular resolution with spatial CITE-seq. Nat Biotechnol. (2023) 41:1405–9. doi: 10.1038/s41587-023-01676-0, PMID: 36823353 PMC10567548

[48] Zhang JP Song Z Wang HB Lang L Yang YZ Xiao W . A novel model of controlling PD-L1 expression in ALK(+) anaplastic large cell lymphoma revealed by CRISPR screening. Blood. (2019) 134:171–85. doi: 10.1182/blood.2019001043, PMID: 31151983 PMC6624970

[49] Xu HC Wang R Shinde PV Walotka L Huang A Poschmann G . Slow viral propagation during initial phase of infection leads to viral persistence in mice. Commun Biol. (2021) 4:508. doi: 10.1038/s42003-021-02028-x, PMID: 33927339 PMC8084999

[50] Duhan V Khairnar V Kitanovski S Hamdan TA Klein AD Lang J . Integrin alpha E (CD103) limits virus-induced IFN-I production in conventional dendritic cells. Front Immunol. (2020) 11:607889. doi: 10.3389/fimmu.2020.607889, PMID: 33584680 PMC7873973

[51] Schulze H Kolter T Sandhoff K . Principles of lysosomal membrane degradation: Cellular topology and biochemistry of lysosomal lipid degradation. Biochim Biophys Acta. (2009) 1793:674–83. doi: 10.1016/j.bbamcr.2008.09.020, PMID: 19014978

[52] Ali S Hoven A Dress RJ Schaal H Alferink J Scheu S . Identification of a novel Dlg2 isoform differentially expressed in IFNbeta-producing plasmacytoid dendritic cells. BMC Genomics. (2018) 19:194. doi: 10.1186/s12864-018-4573-5, PMID: 29703139 PMC6389146

