# Supplementary material for: Acid ceramidase regulates CD8+ T-cell exhaustion via type I interferon-mediated upregulation of PD-L1
Source: Front Immunol. 2025 Dec 9;16:1638403. doi: 10.3389/fimmu.2025.1638403 (PMC12722844; doi:10.3389/fimmu.2025.1638403)
Supplement: Supplementary file 1 [file Presentation1.pdf]

Supplementary: Acid ceramidase regulates CD8$^+$ T cell exhaustion via Type I interferon mediated upregulation of PD-L1

Supplementary Table 1

Figure 1A legend	Cell Type	GEO accession	Abbreviation	Sequencing reads of *Asah1*	Sequencing reads of GAPDH	Relative expression (*Asah1* /GAPDH)
T4	splenic TCRb+CD4+CD8- T cells	GSM1945034	T4.SP.SPF#1	343.9434	7393.305829	0.046520921
		GSM1945035	T4.SP.SPF#2	328.288	7008.131243	0.046843872
		GSM1945078	T4.SP.SPF#3	190.939	5762.582133	0.033134278
T8	splenic TCRb+CD4-CD8+ T cells	GSM1945036	T8.SP.SPF#1	327.4418	7373.15965	0.04440997
		GSM1945037	T8.SP.SPF#2	284.4667	7313.832048	0.038894344
		GSM1945079	T8.SP.SPF#3	203.8725	6150.459986	0.033147521
B	splenic CD19+IgM+ B cells	GSM1945040	BB.SP.SPF#1	556.7529	6092.92365	0.09137697
		GSM1945041	BB.SP.SPF#2	445.7912	5959.671871	0.074801299
		GSM1945084	BB.SP.SPF#3	519.6899	6137.910779	0.084668859
B1	CD19+CD43+CD5 + peritoneal B cells	GSM1945044	B1ab.PC.SPF#1	587.1942	7739.593207	0.075868871
		GSM1945045	B1ab.PC.SPF#2	608.1958	8133.228386	0.074779137
		GSM1945083	B1ab.PC.SPF#3	459.7588	8557.158888	0.053727973
TR	TCRb+CD4+CD2 5+ Treg cells	GSM1945046	Treg.SP.SPF#1	373.4189	8581.509402	0.043514361
		GSM1945047	Treg.SP.SPF#2	378.5732	8515.598517	0.044456441
		GSM1945080	Treg.SP.SPF#3	469.5803	7628.608307	0.061555172
TG	TCRgd+TCRb- T cells	GSM1945048	Tgd.SP.SPF#1	285.0069	5266.681229	0.054115085
		GSM1945049	Tgd.SP.SPF#2	259.1033	6388.16599	0.040559888
		GSM1945081	Tgd.SPF.SPF#3	233.1458	6824.572186	0.034162698
NK	splenic NK1.1+TCRb- NK cells	GSM1945050	NK.SP.SPF#1	324.257	5832.309021	0.055596677
		GSM1945051	NK.SP.SPF#2	241.4302	5262.356757	0.045878721
		GSM1945076	NK.SP.SPF#3	410.0186	6077.702019	0.067462768
NT	TCRbintNK1.1intN KT cells	GSM1945052	NKT.SP.SPF#1	302.0688	7846.089607	0.03849928
		GSM1945053	NKT.SP.SPF#2	233.2551	8076.42324	0.02888099
		GSM1945077	NKT.SP.SPF#3	441.7036	8278.209026	0.053357387
GN	splenic Ly6C+ neutrophils	GSM1945042	GN.SP.SPF#1	1119.1681	8916.888738	0.125511054
		GSM1945043	GN.SP.SPF#2	1243.6658	9898.78405	0.125638239
DC	splenic CD11c+MHCII+ FLT3+ dendritic cells	GSM1945054	DC.SP.SPF#1	2015.0364	7516.35636	0.268086863
		GSM1945055	DC.SP.SPF#2	2079.3135	7499.524907	0.277259363
		GSM1945082	DC.SP.SPF#3	2074.5824	7051.731131	0.294194767
MF	F4/80+ICAM2+ peritoneal cavity macrophages	GSM1945038	MF.PC.SPF#1	2012.4113	9704.327448	0.207372568
		GSM1945039	MF.PC.SPF#2	2313.344	11880.85539	0.194711906
		GSM1945085	MF.PC.SPF#3	2846.4065	11231.9792	0.253419851

Supplementary Table 1: RNA sequencing original reads data.

Supplementary Figure 1

a

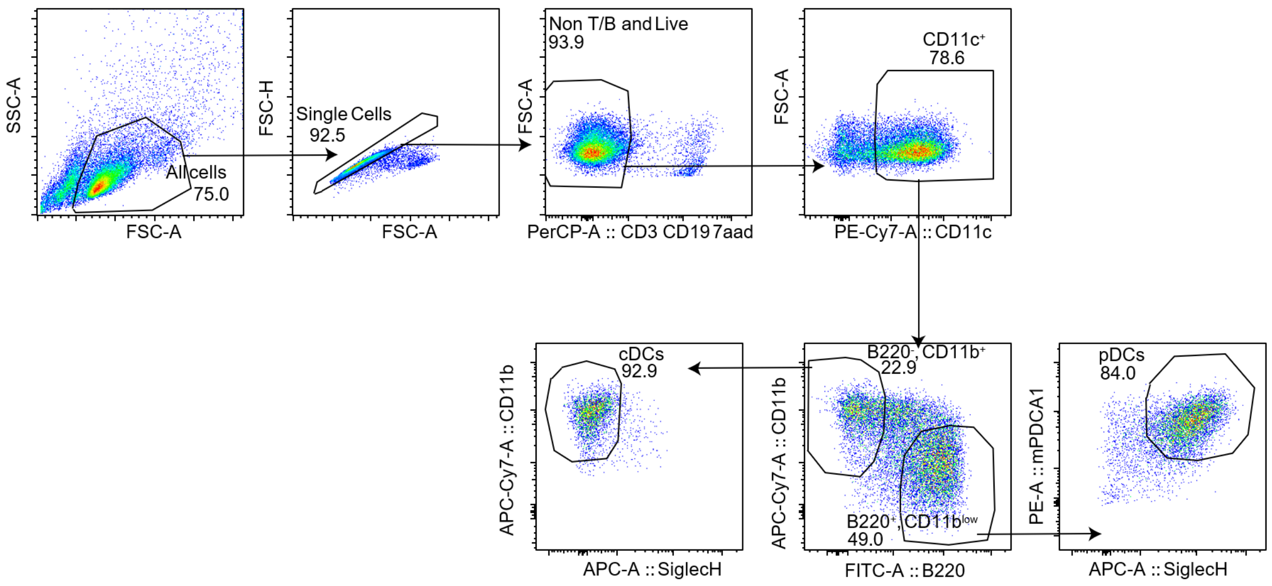

Supplementary Figure 1: **Gating scheme for pDCs and cDCs for *In vitro* BM-Flt3L cultures.** pDCs (40-50%): $CD3^{-}CD19^{-}CD11c^{+}$ $CD11b^{-}$ $B220^{+}$mPDCA-1^{+} SiglecH^{+}. cDCs (20-30%): $CD3^{-}CD19^{-}CD11c^{+}$ $CD11b^{+}$ $B220^{-}$ SiglecH^{-}.

Supplementary Figure 2

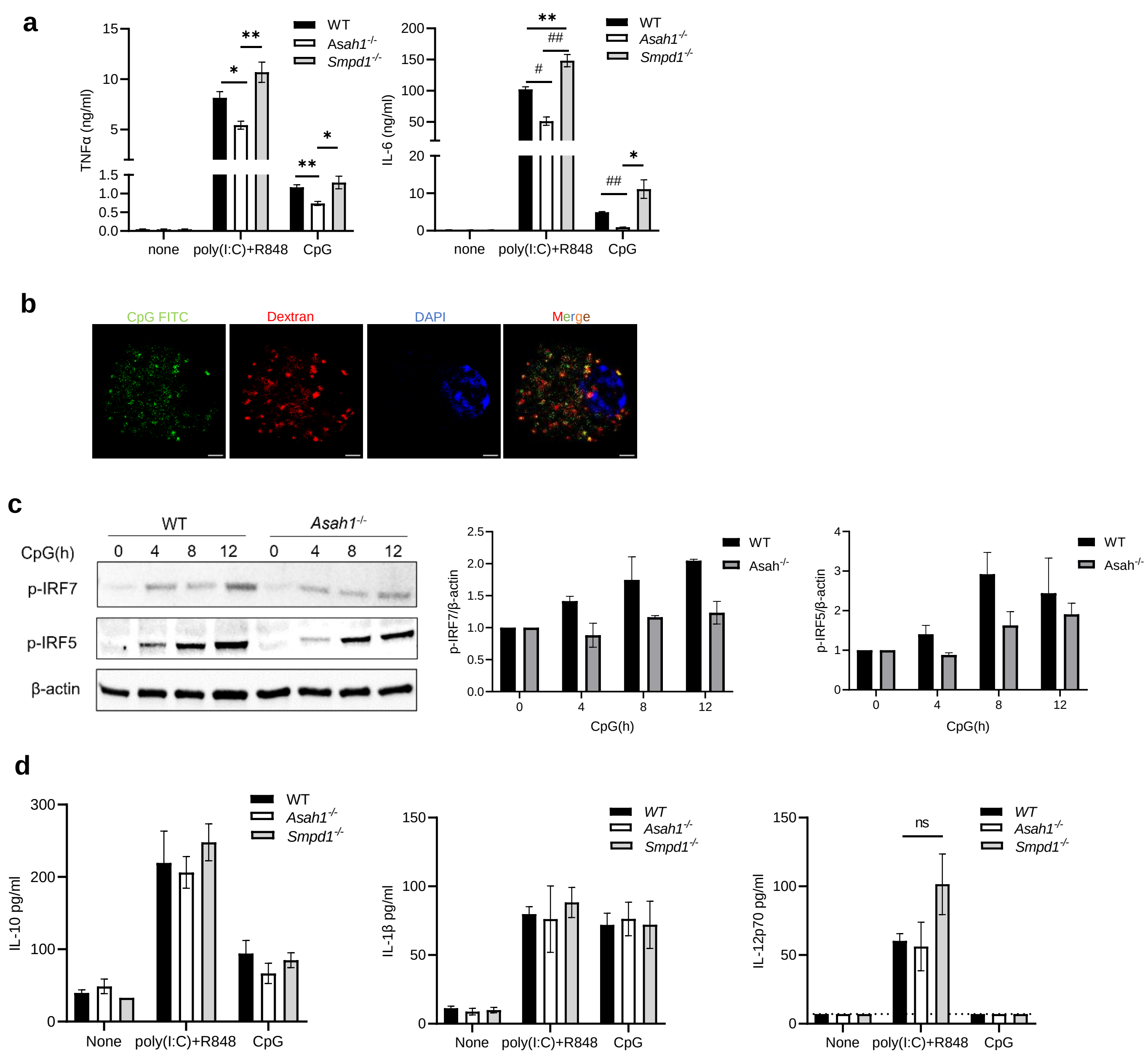

Supplementary Figure 2: Additional evidence that ceramide modulates endosomal nucleic-acid sensing. a ELISA for TNF-α and IL-6 at 16h of supernatants from GM-CSF induced BMDCs stimulated with poly(I:C) (25 µg/ml)+R848 (0.1 µg/ml) or CpG (1 µM; n=6; 2-way ANOVA [Tukey's multiple comparison]). **b** Confocal microscopy of WT Flt3L-DCs incubated with CpG-FITC for 30 min and Alexa Fluor™ 647 labeled Dextran for 10 min before live imaging. Scale bar 5µm. **c** Western Blot (left) densitometric analysis (right) of whole-cell lysates for phospho-IRF5 and phospho-IRF7 of Flt3L-DCs stimulated with Dotap- CpG for 0, 4, 8, 12h, respectively. n=2-3; Data normalized to β-actin expression, presented as fold-change relative to unstimulated WT control. n= 2-3 **d** GM-CSF induced BMDCs were stimulated poly(I:C) (25 µg/ml) +R848 (0.1 µg/ml), CpG (1 µM) for 16h, IL-10, IL-1β and IL-12p70 concentration was determined in the supernatant by ELISA (n=4-6; 2-way ANOVA [Tukey's multiple comparison]). All data are shown as mean +/- SEM. * equals p ≤ 0.05, ** equals p ≤ 0.01, # equals p ≤ 0.001, ## equals p ≤ 0.0001.

Supplementary Figure 3

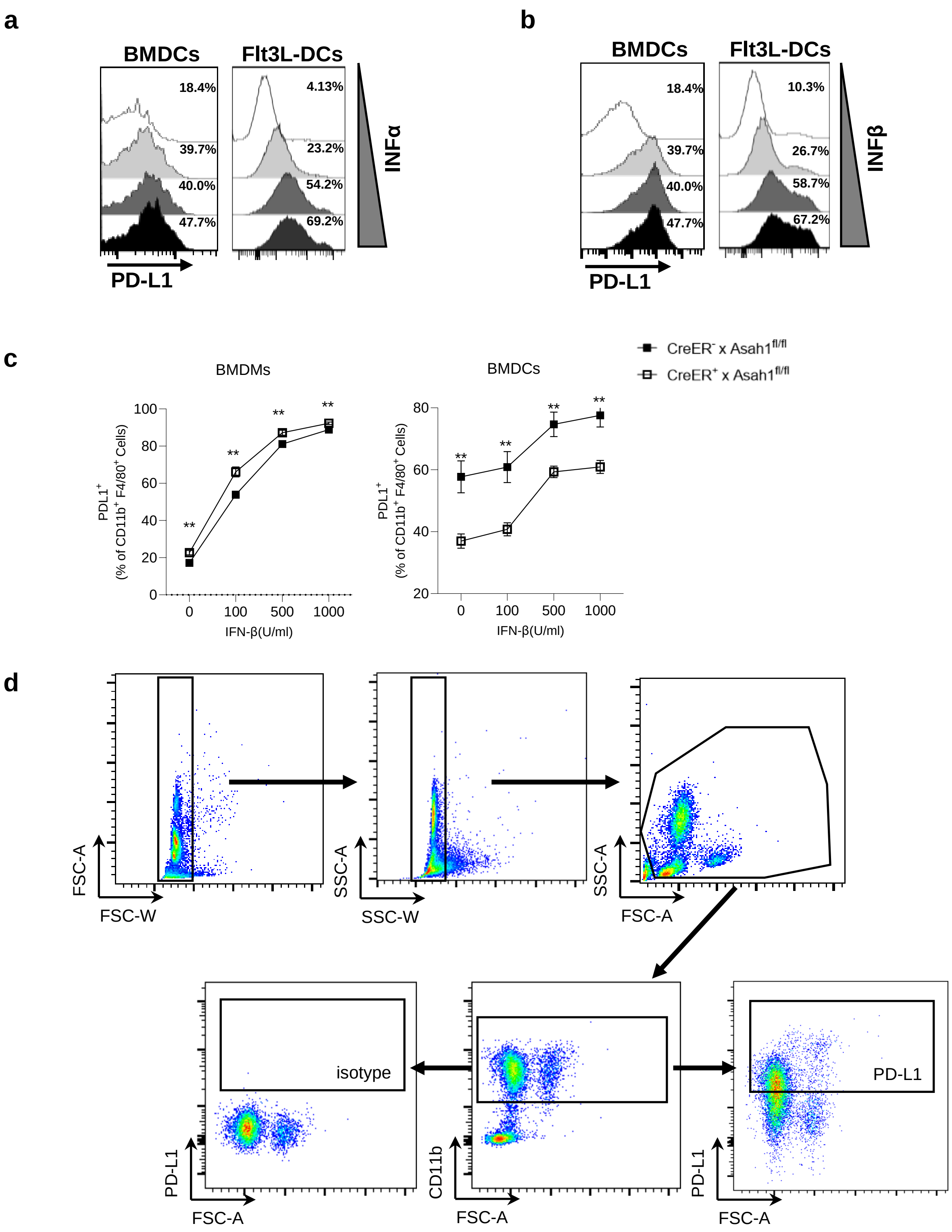

Supplementary Figure 3: | **IFN-β-induced PD-L1 expression in WT and Asah1-deficient APCs a-b** GM-CSF induced bone marrow derived dendritic cells (BMDCs) and Flt3L induced bone marrow derived dendritic cells (Flt3L-DCs) from C57BL/6 wildtype (WT) mice were treated by recombinant mouse IFNα (**a**) and IFNβ (**b**) protein with 0, 100, 500, 1000U/ml for 24h. Histogram of PD-L1 (CD274+) expression was determined by flow cytometry. **C** PD-L1 expression (% positive) measured by flow cytometry on WT (CreER- × *Asah1*$^{fl/fl}$) and KO (CreER+ × *Asah1*$^{fl/fl}$) BMDMs (left) and GM-CSF-derived BMDCs (right) stimulated with IFN-β (0, 100, 500, 1000U/ml) for 24 h . Mean ± SEM; n =3 biological replicates each in duplicates. Unpaired student's t-test. **d** Gating strategy of PD-L1 staining.

Supplementary Figure 4

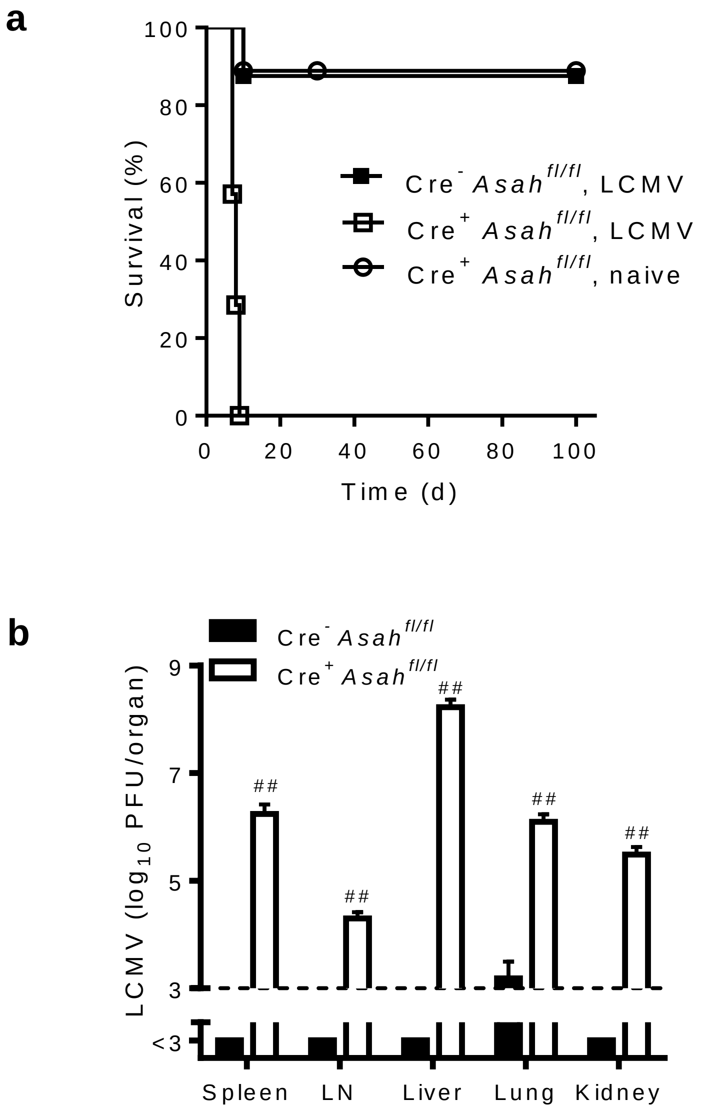

Supplementary Figure 4: **Virus persistence and death in aCDase-deficient mice. a** Survival of tamoxifen-treated Cre⁻ *Asah1*$^{fl/fl}$ and tamoxifen-treated Cre⁺ *Asah1*$^{fl/fl}$ mice that were infected intravenously with 2×10^4 plaque-forming units (PFU) LCMV-WE (n = 7-8), or tamoxifen-treated Cre⁺ *Asah1*$^{fl/fl}$, that were not infected (n = 9), p ≤ 0,0001. **b** Plaque assay of various organs from tamoxifen-treated Cre⁻ *Asah1*$^{fl/fl}$ and Cre⁺ *Asah1*$^{fl/fl}$ mice that were infected intravenously with 2×10^4 PFU LCMV-WE and analyzed on day 8 after infection (n = 6-10). All data are shown as mean +/- SEM. * equals p ≤ 0,05, ** equals p ≤ 0,01, # equals p ≤ 0,001, ## equals p ≤ 0,0001.

Supplementary Figure 5

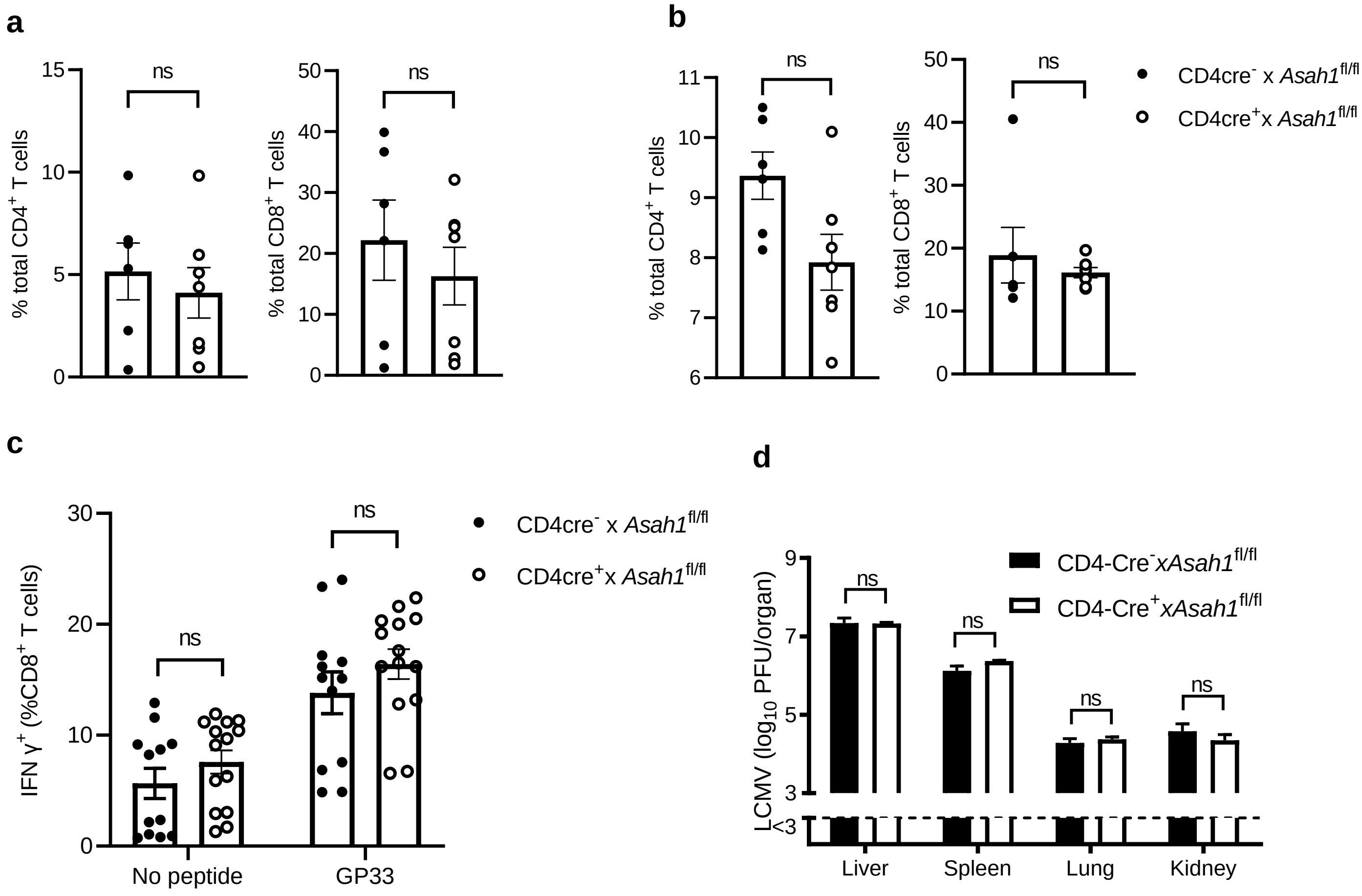

Supplementary Figure 5: **aCDase of pDCs but not T cells is required for efficient immune response**. Tamoxifen-treated CD4-Cre^{-} *Asah1$^{fl/fl}$* and tamoxifen-treated CD4-Cre^{+} *Asah1$^{fl/fl}$* mice that were infected intravenously with 2×10^{6} plaque-forming units (PFU) LCMV-Docile (n = 4-8) and analysis was performed on day 8 post infection (p.i.). **a&b** Percentage of CD4^{+} (left) and CD8^{+} (right) cells in lymphocytes from blood (**a**) and spleen (**b**). **c** Frequency of IFN-γ^{+} non- or GP33 restimulated CD8^{+} T cells. **d** viral titers in various organs. All data are shown as mean +/- SEM; ns = non significant.
